# Fast, 3D Isotropic Imaging of Whole Mouse Brain Using Multiangle‐Resolved Subvoxel SPIM

**DOI:** 10.1002/advs.201901891

**Published:** 2019-12-03

**Authors:** Jun Nie, Sa Liu, Tingting Yu, Yusha Li, Junyu Ping, Peng Wan, Fang Zhao, Yujie Huang, Wei Mei, Shaoqun Zeng, Dan Zhu, Peng Fei

**Affiliations:** ^1^ School of Optical and Electronic Information‐Wuhan National Laboratory for Optoelectronics Huazhong University of Science and Technology Wuhan 430074 China; ^2^ Britton Chance Center for Biomedical Photonics Wuhan National Laboratory for Optoelectronics Huazhong University of Science and Technology Wuhan 430074 China; ^3^ MoE Key Laboratory for Biomedical Photonics Huazhong University of Science and Technology Wuhan 430074 China; ^4^ Department of Anesthesiology Tongji Hospital Tongji Medical College Huazhong University of Science and Technology Wuhan 430030 China

**Keywords:** brain imaging, computational imaging, light‐sheet fluorescence microscopy, neuroscience, super resolution

## Abstract

The recent integration of light‐sheet microscopy and tissue‐clearing has facilitated an important alternative to conventional histological imaging approaches. However, the in toto cellular mapping of neural circuits throughout an intact mouse brain remains highly challenging, requiring complicated mechanical stitching, and suffering from anisotropic resolution insufficient for high‐quality reconstruction in 3D. Here, the use of a multiangle‐resolved subvoxel selective plane illumination microscope (Mars‐SPIM) is proposed to achieve high‐throughput imaging of whole mouse brain at isotropic cellular resolution. This light‐sheet imaging technique can computationally improve the spatial resolution over six times under a large field of view, eliminating the use of slow tile stitching. Furthermore, it can recover complete structural information of the sample from images subject to thick‐tissue scattering/attenuation. With Mars‐SPIM, a digital atlas of a cleared whole mouse brain (≈7 mm × 9.5 mm × 5 mm) can readily be obtained with an isotropic resolution of ≈2 µm (1 µm voxel) and a short acquisition time of 30 min. It provides an efficient way to implement system‐level cellular analysis, such as the mapping of different neuron populations and tracing of long‐distance neural projections over the entire brain. Mars‐SPIM is thus well suited for high‐throughput cell‐profiling phenotyping of brain and other mammalian organs.

## Graphical abstract

The newly developed multiangle‐resolved subvoxel selective plane illumination microscope (Mars‐SPIM) can rapidly image whole mouse brain at isotropic single‐cell resolution, on a timescale of minutes. Through computationally restoring the degraded signals from deep tissue and improving the spatial resolution under a very large field of view, Mars‐SPIM shows great potential for high‐throughput, high‐resolution mapping of entire large organs/organisms.

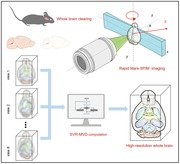

## 1 Introduction

The comprehensive understanding of complex cellular connections in the whole mammalian brain is one of the fundamental challenges in neuroscience. To unravel the various neuronal profiles of different physiological functions in the whole brain, 3D high‐resolution (HR) imaging is required over a mesoscale sized volume.^1^ However, creating such a large‐scale brain dataset has posed a big challenge for current 3D optical microscopy methods, all of which show relatively small optical throughputs.^2^, ^3^ Furthermore, light scattering and attenuation are outstanding issues for the turbid tissues that limit the extraction of signals from deep brain. To address these issues, 3D tile stitching combined with brain sectioning has been a popular strategy for obtaining mammalian brain atlases, which can be a meaningful platform for mapping neuronal populations, activities, or connections over the entire brain.^4^ For example, sequential two‐photon tomography (STPT) can 3D image the brain at subcellular high resolution,^5^, ^6^ but at the cost of long acquisition times of up to several days and a high‐maintenance system setup. The advent of light‐sheet fluorescence microscopy^7^ (LSFM) in conjunction with tissue‐clearing^8^ eliminates the need for complicated mechanical slicing of samples by instead applying nondestructive optical sectioning. Although LSFM still needs repetitive image stitching to achieve high lateral resolution over a large field of view (FOV), its use of wide‐field detection results in higher imaging speeds compared with the point‐by‐point scanning of epifluorescence methods. A few well‐known derivations of LSFM, for example, selective plane illumination microscopy (SPIM), have recently been used for mouse brain imaging with balanced speed and spatial resolution. However, the axial extent of the plane illumination in SPIM has to be compromised to its lateral illumination FOV so that an anisotropic axial resolution (typically of 5–20 µm, depending on the size of the mosaic patch needing to be illuminated) can be yielded for whole‐brain scale imaging.^9^, ^10^ As a result, it is difficult to resolve fine neuronal structures and connections in 3D, as can be achieved by conventional epifluorescence methods such as micro‐optical sectioning tomography^11^ and STPT.^6^ Furthermore, even with the much larger imaging depth enabled by tissue clearing,^10^, ^12^ photon absorption and scattering still occur in the clarified tissues of whole mammalian organs. These cause noticeable deterioration of signals from deep tissues. Recently, multiview fusion techniques,^13^, ^14^ which have previously been used in the imaging of small live embryos,^15^ have also been applied to excised mouse brains. These can improve the relatively low axial resolution and suppress deep tissue scattering.^16^ However, for the direct imaging of mesoscale intact organs, the lateral resolution of SPIM systems, being 5 µm at its best, is insufficient to visualize single cells under a large FOV of over 5 mm. In such circumstances, multiview techniques cannot overcome the lateral resolution limit determined by the detection optics. However, if multiview techniques were to be combined with repetitive image stitching, the throughput advantage of LSFM would be significantly reduced as well as the photon utility.

Here, we present a whole‐brain mapping pipeline, termed multiangle‐resolved subvoxel SPIM (Mars‐SPIM), which can image the whole mouse brain at an isotropic voxel resolution of ≈1 µm with a high throughput rate of half an hour per brain. This imaging strategy combines our subvoxel‐resolving (SVR) computation^17^ with multiview Bayesian deconvolution (MVD)^14^, ^18^ to achieve fast and accurate reconstruction of a whole brain with isotropically improved resolution. Unlike conventional whole‐brain imaging methods that use stepwise *z*‐scanning and 3D tile stitching, Mars‐SPIM directly records low‐resolution (LR) whole‐brain images using a continuous nonaxial scanning method. This unique scanning mode provides a high acquisition rate, and meanwhile encrypts subresolution shifts into raw images, which could be further processed by the multiview subvoxel‐resolving computation. Furthermore, this computation pipeline is parallelized with multi‐graphics processing units (GPUs) to achieve a high reconstruction throughput (gigavoxels per minute) that matches the fast image acquisition. By reconstructing a single‐cell‐resolution whole‐brain atlas, we demonstrate successful brain‐wide tracing of single neural projections and the counting of different neuronal populations over the entire brain. Mars‐SPIM shows spatial‐temporal performance superior to the other available techniques for large‐scale cell profiling, where sample size, spatial resolution, and imaging throughput are all highly valued. It is therefore suitable for system‐level cellular analysis of brain or other whole organs.

## 2 Results

### 2.1 Mars‐SPIM Setup and Characterization

**Figure 1:**
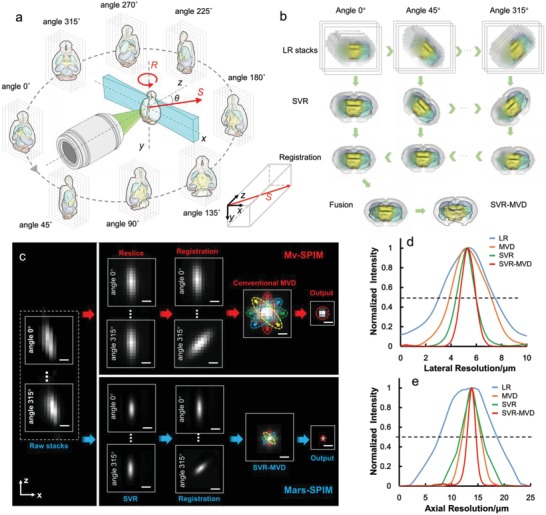
Illustration and characterization of Mars‐SPIM. a) The schematic of Mars‐SPIM. A low NA objective generates a relatively thick light‐sheet and a low‐magnification objective collects fluorescence with large FOV. The operation of the sample includes four degrees of freedom as: *x*‐axis, *y*‐axis, nonaxial direction S, and rotation (along *y*‐axis). Unlike standard *z*‐scan SPIM, here the 3D scanning vector S (red) has a certain angle θ with respect to the *x*, *y*, *z* axes. A coordinate is shown at the bottom right. This off‐*z*‐axis scanning strategy in conjunction with a small step size encrypts the raw image stack with lateral and axial subvoxel‐size shifts, which can be used to reconstruct a resolution‐enhanced volumetric image via SVR procedure. To suppress the light scattering from the deep tissues and achieve isotropic 3D resolution, the whole brain sample is rotated and imaged under eight views. b) The work flow of SVR‐MVD procedure which can in toto reconstruct the whole brain at isotropically enhanced resolution. It majorly includes: first, the SVR computation for multiview, subvoxel‐scanned raw images; second, feature‐based registration of SVR‐processed images; and third, a Bayesian‐based deconvolution that generates the final output based on multiview SVR images. c) The resolution comparison between single‐view raw image, SVR only, MVD only and SVR‐MVD, via resolving subresolution fluorescent beads (≈500 nm diameter). *x*–*z* images show the lateral and axial extents of the resolved beads (red circles). d,e) The intensity plots of the linecuts through the resolved beads along the lateral and axial directions, respectively. The SVR‐MVD shows an obviously highest isotropic resolution at ≈1.4 µm, which is compared to ≈4.2 (lateral) and 12 µm (axial) in raw image. Scale bars: 5 µm in (c).

We developed a Mars‐SPIM system, with a low‐profile setup with wide‐FOV illumination and detection sufficient to cover the entire mouse brain (see the Experimental Section and Figures S1 and S2, Supporting Information). Under a certain view, the brain sample to be imaged is continuously scanned across the laser sheet along a nonaxial direction (vector S in **Figure** **1**). The camera is synchronized to sequentially record a stack of plane images with a step‐size significantly smaller than the laser‐sheet thickness. This nonaxial scanning mode of the Mars‐SPIM encodes the LR raw image stack with subvoxel spatial shifts, which are then used to reconstruct HR images through the application of an SVR algorithm (Figure S3, Supporting Information). The sample is then rotated along the *y*‐axis and nonaxially imaged under multiple views (Figure 1a). After the SVR computation has generated a series of HR images with anisotropic volumes for all the views, a neuron‐feature‐based registration followed by a MVD procedure is applied to accurately align the multiple SVR volumes, compute their conditional probabilities, and finally produce an output with recovered complete signals and isotropic super‐resolution in 3D. This SVR‐MVD fused computation pipeline is further illustrated in Figure 1b and Figure S3 in the Supporting Information.

We used fluorescent microbeads with a diameter of ≈500 nm as a point source to characterize the Mars‐SPIM system (Figure 1c and Figure S4, Supporting Information). For each view, the microbeads were scanned by a ≈12 µm thick (full width at half maximum value, FWHM) laser sheet with 280 nm step size and were detected by a 4×/0.16 objective. This nonaxial scanning process (10° to the *x*–*z* and *y*–*z* planes) generated subresolution shifts of 48 and 272 nm in the lateral and axial directions respectively. Thirty‐four groups of LR image volumes representing the standard resolution of the system optics (voxel size: 1.625 µm × 1.625 µm × 4.5 µm) were extracted from the raw image sequence to compute the HR images. The raw LR, single‐view SVR, conventional MVD, and SVR‐MVD results are compared in Figure 1c. The line intensity profiles of the resolved beads are plotted in Figure 1d,e to indicate the lateral and axial resolutions of these methods. The achievable lateral and axial FWHMs of the SVR‐MVD are improved from ≈4.2 µm and 12 µm in the raw image to isotropic values of ≈1.4 µm, which are superior to both the single‐view SVR (1.7 and 4.5 µm) and conventional MVD (isotropic 3.4 µm).

**Figure 2:**
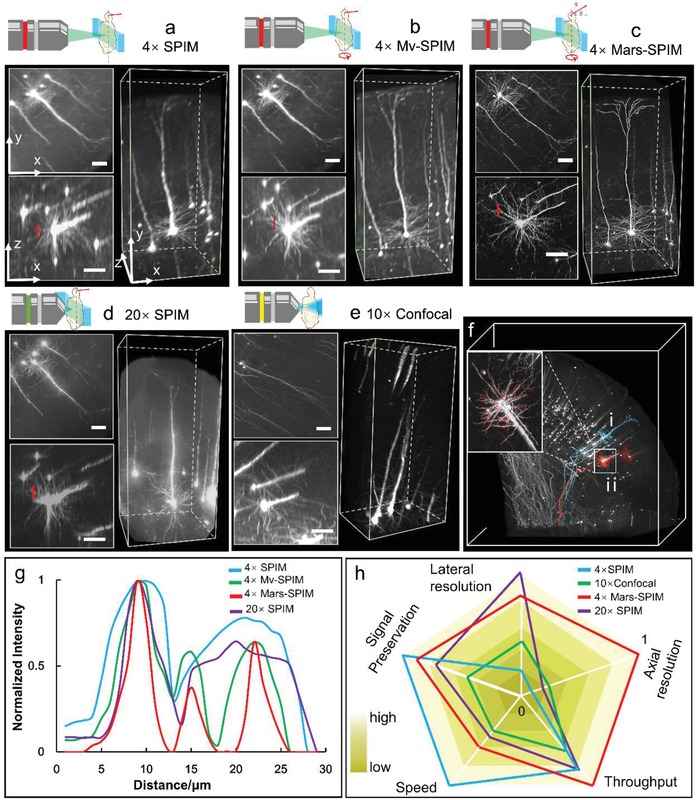
Mars‐SPIM demonstration on *thy1‐GFP‐M* brain block. a) Visualization of the neurons at cortex area by conventional SPIM using 4×/0.28 objective plus a 15 µm laser sheet. The voxel size is 1.625 by 1.625 by 6 µm. b) Conventional multiview SPIM (Mv‐SPIM) results with an isotropic voxel size of 1.625 µm × 1.625 µm × 1.625 µm. c) The Mars‐SPIM results of the same neurons, with an isotropic reconstructed voxel size of 0.41 µm × 0.41 µm × 0.41 µm. d) Comparison from high‐resolution SPIM using 20×/0.45 air objective plus 6.5 µm light‐sheet. Due to the increasing spherical aberration under higher magnification, the SNR of the images is obviously decreased. e) Neuron imaging using confocal microscope under 10×/0.4. f) The SVR‐MVD reconstruction of the entire brain block with size around 3 mm by 3 mm by 3 mm. As a result of finer reconstruction, two pyramidal neurons with dendrites and axons are finely segmented, shown as (i) and (ii). Inset shows the vignette high‐resolution view of the segmented neuron (ii) in (f), showing the clearly resolved fibers. g) The intensity plot of the dash lines transversely across a few neural fibers in (a)–(d). It shows that Mars‐SPIM has the narrowest peaks which indicate highest resolving power in practice. h) The radar map that compares the system simplicity, imaging throughput, photobleaching, and spatial resolutions of four methods. The values are outputted by the logarithm and normalized. Scale bars are 50 µm in (a)–(e).

We then demonstrated the imaging capability of the Mars‐SPIM using clarified brain tissue from a transgenic mouse (*thy1‐GFP‐M*). The brain sample was optically sectioned by a 15 µm laser sheet and imaged using a 4×/0.28 objective and a high‐speed camera (Hamamatsu Flash 4.0 v2) at a rate of 50 frames per second. The brain sample was translated at a nonaxial step‐size of 280 nm (4 × 4 × 2 enhancement) and rotated 45° for each new view. Eight different view image stacks were rapidly recorded in a total time of around 20 min. The raw image volume of each view was acquired at the limited resolution of the system optics, and hence the densely packed neuronal fibers remained unresolvable (**Figure** **2**a). The SVR procedure for each view was then started with an initial guess, which was simply a ×4 interpolation of one of the subdivided LR groups, and the process iteratively converged to the HR solution (data not shown). Then, in the multiview registration step, the neuronal cell bodies were recognized as features to establish correspondences, instead of the beads. This cell‐based registration was verified to be as accurate as the bead‐based one (Figures S5 and S6, Supporting Information), while at the same time producing a cleaner visualization (Videos S1 and S2, Supporting Information). Figure 2c shows the final Mars‐SPIM result with a reconstructed voxel size of 0.4 µm. This result is further compared with conventional multiview SPIM (Mv‐SPIM, Figure 2b), high‐magnification SPIM (20×/0.45 with ≈6.5 µm laser sheet, Figure 2d) and confocal microscopy (10×/0.4, Figure 2e). The linecuts through the horizontal plane of the neuron dendrite (Figure 2a–d) using each method reveal significantly improved resolution with the Mars‐SPIM, which surpasses both the 20 × ‐SPIM result with anisotropic resolution in the longitudinal direction, and the Mv‐SPIM result with insufficient overall resolution (Figure 2g). With the substantially enhanced isotropic resolution, two giant pyramidal neurons could be finely segmented across a large volume (≈400 gigavoxels for the entire sample), as shown by the blue and red colors in Figure 2f,g. We note that besides the increased space‐bandwidth product (SBP; volume size divided by resolution),^3^ the Mars‐SPIM also shows an improvement in the signal‐to‐noise ratio (SNR), which can help to discern weak signals from the strong background signal of thick tissue (Figure S7, Supporting Information). Furthermore, we tested different numbers of views to verify that eight views formed a good balance between the data size/throughput and performance (Figure S8, Supporting Information). Mars‐SPIM can thus be considered as a light‐sheet microscope that is less vulnerable to spherical aberration and light scattering in thick tissue and combines a large FOV with high‐resolution advantages that are difficult to achieve with previous methods. From another perspective, the stitching‐free continuously scanning mode exhibits a higher acquisition throughput than other stitching‐based methods as well as lower photobleaching. In Figure 2h, we rate the imaging performances of standard 4 × SPIM, 20 × SPIM, 10 × confocal, and our 4 × Mars‐SPIM through comparisons of the system complexities, imaging speeds, photo‐bleaching rates, and spatial resolutions (also see Figure S9 and Table S1, Supporting Information). Compared with the confocal microscope (10×), the Mars‐SPIM gains advantages in imaging depth and axial resolution (Figures S10 and S11, Supporting Information). Besides the well‐balanced volumetric resolution, the Mars‐SPIM yields the highest effective throughput at ≈400 gigavoxels SBP in a 20 min acquisition. Mars‐SPIM also eliminates the need for mechanical stitching, slicing, high‐maintenance optics, and precisely modulated illumination, instead using a relatively simple light‐sheet setup and fast GPU‐based computation to address the general challenge of high‐throughput high‐resolution 3D microscopy that was originally coupled to the physical limitations of a system's optics. In the following whole‐brain applications, this underlying robustness allows the Mars‐SPIM prototype to image the entire thick organ with high spatial‐temporal performance while maintaining a simple setup.

### 2.2 High‐Throughput, In Toto Imaging of Whole Mouse Brain at High Resolution

**Figure 3:**
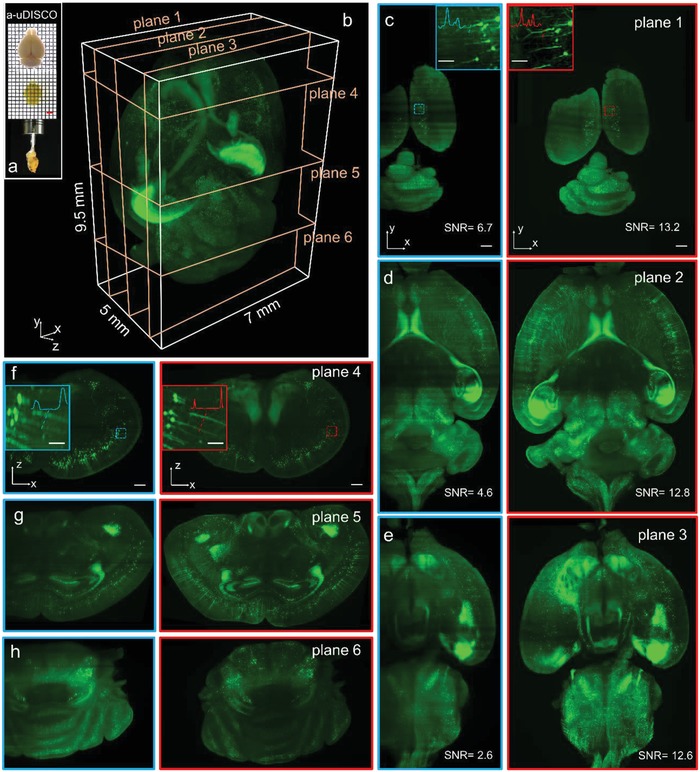
Comparison of whole mouse brain image by conventional SPIM and Mars‐SPIM. a) The photographs of an adult mouse brain (8 weeks) before and after a‐uDISCO clearing. b) The 3D reconstruction of cleared whole mouse brain. With obtaining optically cleared brain for light‐sheet imaging, our Mars‐SPIM system rapidly provides 3D visualization of entire brain via SVR‐MVD reconstruction (400 gigavoxels). Under each view, the sample is imaged using 2.2× magnification plus ≈25 µm laser sheet. The subvoxel scanning step size is ≈1 µm. The final result is recovered from raw images of eight views, with reconstructed isotropic voxel size of 1 µm by 1 µm by 1 µm. The imaging throughput here is ≈30 min per whole brain, and the postprocessing time is ≈12 h with employing quad NVIDIA graphical cards. c–e) Compare the horizontal (*xy*) planes (shown in (b)) at 500, 2500, and 4000 µm *z*‐depth, by conventional SPIM (blue outlines), and Mars‐SPIM (red outlines). Mars‐SPIM provides obviously more uniform image quality across the depth of tissue, showing higher and more stable SNR values at all *z*‐depths. f–h) Correspondingly compare the reconstructed coronal (*xz*) planes (shown in (b)) at the height of 1500, 4000, and 8000 µm. During image acquisition under each view, the completely blurred parts (leftmost regions in 0° SPIM image) by tissue scattering/attenuation are discarded for faster imaging, less SVR computation, and better effect of MVD. Besides the reconstruction integrity of whole brain, the insets in (c)–(e) and (f)–(g) further compare the achieved lateral and axial resolutions of regular SPIM images and Mars‐SPIM images with using the same optics. Scale bars: 500 µm in (b)–(g) and 100 µm in insets.

**Figure 4:**
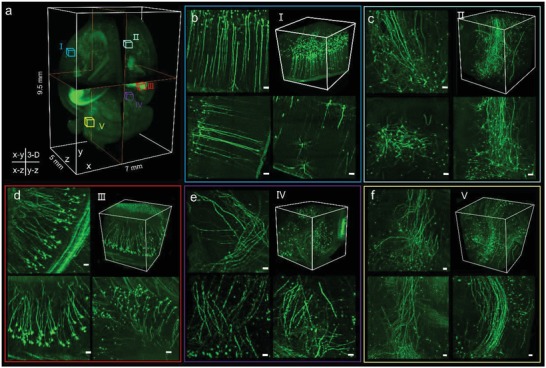
High‐throughput, whole‐brain imaging at isotropic cellular resolution using Mars‐SPIM. a) The reconstructed whole mouse brain by Mars‐SPIM. Five selected volumes (I–V) at left cortex (blue), right cortex (cyan), hippocampus (red), thalamus (purple), and cerebellum (yellow) are shown in (b)–(f), respectively, with each one containing the horizontal (*x*–*y*), sagittal (*y*–*z*), and coronal (*x*–*z*) planes and 3D rendering of the selected volume. The neuronal cell bodies together with the projecting fibers can be identified as a result of significantly enhanced resolutions by Mars‐SPIM. Scale bars: 20 µm in (b)–(f).

An 8‐week‐old whole mouse brain (*Tg*: *thy1‐GFP‐M*) was optically cleared using the a‐uDISCO method,^19^ before being imaged by the Mars‐SPIM. The brain shrank in size from ≈9.3 mm × 14 mm × 7.1 mm to ≈7 mm × 9.5 mm × 5 mm after clearing (**Figure** **3**a). Despite the use of tissue clearing, light attenuation/scattering from deep tissue remained a big challenge for the complete imaging of the whole brain (Figure S13, Supporting Information). However, brain‐wide biomedical applications such as cell population mapping and neuronal projection tracing intrinsically need high spatial resolution across a large area, hence highlighting the significance of the Mars‐SPIM method. Experimentally, the whole brain was imaged under a low‐magnification setup of 25 µm light‐sheet illumination plus 2.2 × detection prior to the camera detection and therefore only required to be stitched twice because of the large FOV. The brain was then rotated for each view of the nonaxial scanning (950 nm step size, 5500 frames in 110 s), with a total of 16 views of raw stacks being rapidly obtained in around half an hour. Using the above mentioned SVR‐MVD procedure, we successfully reconstructed the entire brain at an isotropic voxel size of 0.975 µm. Figure 3b shows a reconstructed volume rendering of the whole brain (400 gigavoxels, maximum intensity projection mode). The horizontal planes (*x*–*y*) at different depths (Figure 3c–e) and coronal planes (*x*–*z*) at different heights (Figure 3f–h) are extracted from the reconstructed brain volume and compared with the conventional SPIM results. It is obvious that the Mars‐SPIM shows remarkable improvements in resolution, contrast, and signal integrity. Vignettes of high‐resolution Mars volumes of five selected areas (**Figure** **4**a), including the left and right cortex, hippocampus, thalamus, and cerebellum, are shown in Figure 4b–f, respectively. The strong efficacy of the neural signal recovery as well as the resolution enhancement by Mars‐SPIM is further illustrated in Figure S13 in the Supporting Information, in which a full coronal plane acquired by conventional SPIM and experiencing strong scattering from both illumination (*x*) and emission (*z*) is compared with the same plane acquired by Mars‐SPIM. By quickly creating a cellular‐resolution brain atlas encompassing 400 gigavoxels across a large volume of over 300 mm^3^ (postcomputation time ≈12 h), the Mars‐SPIM enables high‐throughput analysis of massive neurons at the whole brain level, which are otherwise spatially or temporally more challenging using regular light sheet microscopes. (Figure 2h and Figure S14, Supporting Information).

### 2.3 Whole‐Brain Visualization and Segmentation

**Figure 5:**
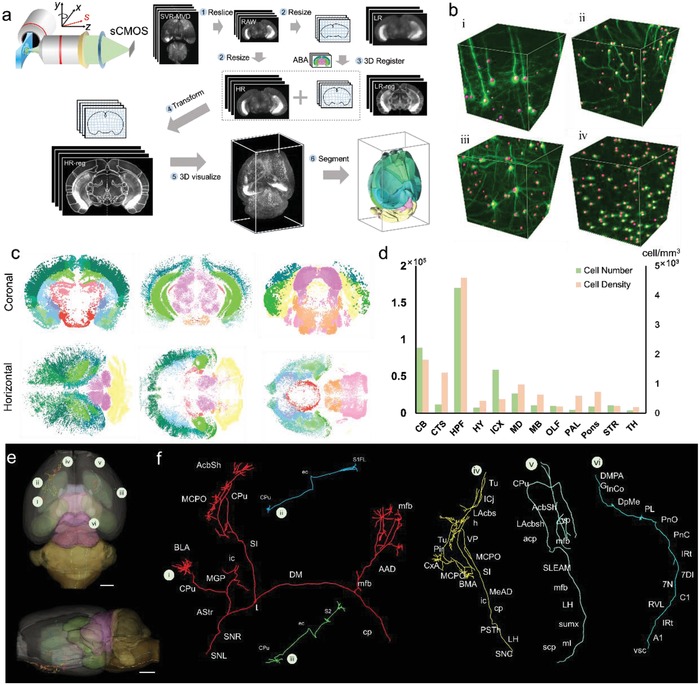
Quantifications of a *thy1‐GFP‐M* mouse brain based on Mars‐SPIM image. a) The quick creation of whole brain atlas. Step 1: Reorientation of the Mars‐SPIM image from horizontal to coronal view and prealignment to standard Allen brain atlas (ABA) using Elastix. Step 2: Resizing the prealigned coronal images into low‐resolution (LR) and high‐resolution (HR) groups. Step 3: Registration of LR group to ABA to obtain the transformation matrix. Step 4: Application of the transformation matrix to HR group to obtain the registered HR images. Step 5: 3D visualization of the ABA‐registered brain. Step 6: Segmentation of the brain regions in Amira. b) With isotropic single‐cell resolution at whole‐brain scale, 3D detection of single neurons can be readily achieved at various brain regions. As a result, the neuron distribution at different regions of the whole brain can be mapped out, as shown in the coronal and horizontal views in (c). Each color represents a brain region. d) The neuron population and density calculated at 12 primary brain regions. CB, cerebellum; CTS, cortical subplate; HPF, hippocampal formation; HY, hypothalamus; ICX, isocortex; MD, medulla; MB, midbrain; OFL, olfactory areas; PAL, pallidum; Pons, pons; STR, striatum; TH, thalamus. e) Horizontal and coronal views of the traced neuron long‐distance projections shown in volumetric rendering, scale bars: 1 mm. f) The pathway annotations of six long‐distance projection neurons. Abbreviations: AAD, anterior amygdaloid area, dorsal part; Acbsh, accumbens nucleus, shell; Astr, amygdalostriatal transition area; BLA, basolateral amygdaloid nucleus, anterior part; cp, CPu, caudate putamen (striatum); DM, ic, LH, lateral hypothalamic area; MCPO, magnocellular preoptic nucleus; mfb, medial forebrain bundle; MGP, medial globus pallidus (entopeduncular nucleus); SI SNL, substantia nigra, lateral part; SNR, substantia nigra, reticular part; Tu, olfactory tubercle; ICj, islands of Calleja; LAcbsh, lateral amygdaloid nucleus; VP, ventral pallidum; Pir, piriform cortex; CxA, cortex‐amygdala transition zone; SI, substantia innominate; BMA, basomedial amygdaloid nucleus, anterior part; MeAD, medial amygdaloid nucleus, anterior dorsal; ic, internal capsule; cp, cerebral peduncle, basal part; PSTh, parasubthalamic nucleus; LH, lateral hypothalamic area; SNC, substantia nigra, compact part; vsc, ventral spinocerebellar tract; A1, A1 noradrenaline cells; IRt, intermediate reticular nucleus; RVL, rostroventrolateral reticular nucleus; C1, C1 adrenaline cells; 7N, facial nucleus; 7DI, facial nucleus, dorsal intermediate subnucleus; PnC, pontine reticular nucleus, caudal part; PnO, pontine reticular nucleus, oral part; PL, paralemniscal nucleus; DpMe, deep mesencephalic nucleus; InCo, intercollicular nucleus; DMPAG, dorsomedial periaqueductal gray; S1FL, primary somatosensory cortex, forelimb region; CPu, caudate putamen; ec, external capsule; S2, secondary somatosensory cortex; scp, superior cerebellar peduncle; ml, medial lemniscus; sumx, supramammillary decussation; LH, lateral hypothalamic area; mfb, medial forebrain bundle; SLEAM, sublenticular extended amygdala, medial part; acp, anterior commissure, posterior; AcbSh, accumbens nucleus, shell; vp, ventral pallidum.

Using the Mars‐SPIM reconstruction of the whole brain (8‐week‐old mouse), we explored the neuronal cyto‐structures in various brain subregions and precisely traced the interregional long‐distance (LD) projections of neurons which is crucial for understanding the functionality of the brain (**Figure** **5** and Videos S3–S5, Supporting Information). After the Mars‐SPIM reconstruction of the whole brain, we used an adaptive registration method^20^, ^21^ to 3D map the brain to the standard Allen Brain Atlas (ABA). The brain was first reorientated from horizontal view to coronal view and automatically prealigned to the ABA using Elastix.^20^ This prealigned brain was then resized into LR and HR groups, as shown in Figure 5a, step 2. Next, we finely registered the LR group to the ABA and obtained the transform correspondence (step 3), which was then applied to the HR group to obtain a registered and transformed HR brain (step 4). This mapped brain atlas was finally visualized in Imaris to facilitate the neuron analysis (Figure 5a).

With the creation of the atlas, the neurons localized to different encephalic regions (such as cortex, hippocampus, cerebellum, and midbrain) could be identified (Figure 5b) and in toto mapped out at a whole‐brain scale (Figure 5c). Then, the neuron population and the density in different encephalic regions were quantified by calculating the volume of the regions and counting the identified cell bodies within them (Figure 5d). The results show that among the 12 primary regions, the hippocampus formation had the highest neuron density of 4600 cells mm^−3^, which is consistent with prior knowledge.^22^ It should be noted that the current low‐number counting results were obtained using a *thy1‐GFP‐M* transgenic mouse, in which GFP signal is expressed by less than 10% of all motor axons, retinal ganglion cells, lumbar dorsal root ganglions, and cortex.^23^ According to the registered HR images, we could trace the neuron projections passing through different brain regions. The whole brain data were volumetrically rendered with several subregions being segmented in different colors. Figure 5e shows horizontal and coronal views of the volume renderings. The trajectories of six LD projection neurons were successfully traced and annotated in the digital whole‐brain, revealing how they were broadcast across the different regions of the brain (Figure 5f). Given the fact that this quantitative analysis was implemented using a *thy1‐GFP‐M* mouse with a large number of neurons being labeled, this procedure should be more efficient if the mouse brain were to be labeled more specifically, such as with a virus tracer. Through the above mentioned demonstration, we have shown the potential of our strategy for imaging‐based quantifications of the whole‐brain, or other whole‐organ‐level analyses, which are crucial for a variety of applications in histology, pathology, and neuroscience.

## 3 Conclusion

Mars‐SPIM can computationally surpass the resolution limit of a regular light‐sheet microscope and suppress the light scattering/attenuation that often exists in thick‐tissue imaging. Unlike mechanical slicing and tile stitching, which require complicated operations, this strategy provides a simple and efficient way to achieve high‐throughput whole‐brain mapping at a single‐cell resolution. The use of simple optics in the Mars‐SPIM offers an ultralarge FOV of hundreds of mm^3^, facilitating direct coverage of the whole brain (or other whole organs). Its stitching‐free continuous scanning mode greatly reduces the acquisition time for such tissue volumes from several hours with traditional methods to several minutes. Complementing the rapid data acquisition, a highly GPU‐parallelized SVR‐MVD computation flow is followed to reconstruct the super‐resolved 3D brain atlas at a high throughput time of a few hours. In our demonstration, the quickly reconstructed digital mouse brain acquired by Mars‐SPIM presents an isotropic cellular resolution (≈2 µm) with three‐ to tenfold improvement in resolution compared with conventional macro‐view SPIM. It should also be noted that this Mars‐SPIM strategy can be applied to most existing light‐sheet microscopes using a simple retrofit and can expand the optical throughput for fast, high‐resolution mapping of whole biological specimens without necessarily increasing the system complexity. Thus, it can be characterized as a high‐throughput 3D imaging method with a simple and cost‐effective setup. Furthermore, the Mars‐SPIM imaging in conjunction with efficient brain registration can form a pipeline for creating an isotropic whole‐brain atlas, with which brain‐wide quantitative analysis (e.g., neuron populations, densities, and long‐distance neuronal projections) could be easily implemented. In combination with recent advances in specimen preparation techniques, such as fluorescence‐friendly tissue clearing, virus‐based sparse labeling, and transgenic animal models, the Mars‐SPIM could be more powerful in enabling various cellular analyses of neural systems. Besides whole‐brain mapping, we believe the Mars‐SPIM method could improve the efficiency of imaging other mammalian organs, such as lung, kidney, and heart, and be of benefit for a wide variety of biomedical applications. Furthermore, its ability to readily accomplish cellular imaging of mesoscale organisms at hundreds of gigavoxel SBP renders Mars‐SPIM a widely applicable tool for cellular profiling, phenotyping, or sample screening assays in histology, pathology, and developmental biology, in which both large‐scale statistics and cellular details are often desired for whole‐tissue‐level study.

## 4 Experimental Section

*Mars‐SPIM Imaging Setup*: A fiber‐coupled diode‐pumped solid‐state laser (CNI laser, 488 nm, single‐mode fiber) was used for excitation source. The laser was first collimated into a Gaussian beam with diameter ≈3.3 mm (Thorlabs, F280FC‐A). Then a sandwich structure containing a convex lens (*f* = 50 mm, Thorlabs, AC254‐050‐A) and two cylindrical lenses (*f* = 30 and 150 mm, Thorlabs, LJ1212L1, LJ1934L1) was designed to transform the round beam into an elliptical shape. The expansion ratio in short (*x*) and long (*y*) axis was ×0.6 and ×3, respectively, forming an elliptical beam with size of 10 mm by 2 mm (Figure S2, Supporting Information). A pair of adjustable mechanical slits (0–8 mm aperture, Thorlabs, VA100C/M) were placed orthogonally to further truncate the beam and thereby tune the height and thickness of the laser sheet. The modulated elliptical beam was equally split into two parts via a 50/50 prism (Thorlabs, CCM1‐BS014/M), to form two opposite beams, which will be further used to illuminate the sample from dual sides. A dual‐side optical sectioning of the whole brain sample was finally formed by using two symmetric combination of cylindrical lens (Thorlabs, LJ1695RM) and illuminating objective (Olympus, 4×/0.10). The laser sheet had a widely tunable range from 5 to 50 µm in thickness and 0.5 to 10 mm in height.

Unlike regular detection setup applied in SPIM, a 4×/0.28 objective was specially used in conjunction with an ED Plan 1× tube lens to construct an infinity‐corrected, wide‐field, and large‐aperture detection path (equivalent magnification ×2.2). Compared to the conventional infinity‐corrected low‐magnification detection, e.g., 2× Nikon objective plus 200 mm focal length tube lens, this setup can collect much more fluorescent signals under a large illumination range due to the larger aperture (Figure S9, Supporting Information). A four‐degree‐of‐freedom motorized stage (*x*, *y*, *z* translation, PT3A/M, Thorlabs; rotation around the *y*‐axis, 42STH38, Phidgets Inc.) integrated with a pair of customized tilting plates (10° inclined surface) was constructed for sample mounting, rotating at multiple angle of views, and scanning across the laser sheet in an off‐detection‐axis direction (Figure S1, Supporting Information). A digital camera (Hamamatsu Orca Flash 4.0 v2 plus, or Andor Zyla 5.5) continuously records the images from the consecutively illuminated planes at a high speed up to 50 full frames per second.

*Ethical Approval*: All animal care and experimental protocols were in accordance with the Experimental Animal Management Ordinance of Hubei Province, P. R. China, and the guidelines of Huazhong University of Science and Technology and were approved by the Institutional Animal Ethics Committee of Huazhong University of Science and Technology.

*Sample Preparation*: Tissue clearing is an essential procedure before imaging. Here a‐uDISCO method was used to clear the brains of 8‐weeks *thy1‐GFP* mice (line M, Jackson Laboratory). In the brain block experiment, to preserve the fluorescence and avoid photobleaching, the cleared brain was embedded into a specific formulated resin^24^ (mixing of D.E.R. 332 (Sigma‐Aldrich, 31185), D.E.R. 736 (Sigma‐Aldrich, 31191), and isophorone diamine, 5‐amino‐1,3,3‐trimethylcyclohexanemethylamine (Sigma‐Aldrich, 118184) at a volume ratio of 4:1:1), the refractive index (1.56) of which was equal to the index‐matched immersion (BABB‐D, mixing of benzyl alcohol (Sigma‐Aldrich), benzyl benzoate (Sigma‐Aldrich), and diphenyl ether (Alfa Aesar) at a volume ratio of 4:8:3). For conducting bead‐based registration, fluorescent beads (Lumisphere, 1% w/v, 500 nm, SiO_2_) were mixed around the sample in the resin. 10 µL of bead stock solution was centrifuged at 1200 rpm for 3 min with the water‐phase supernatant being removed and replaced with 20 µL methanol. Then the methanol‐based bead solution was mixed into the resin to form the bead‐resin mixture, which was finally poured into a tube mold with the brain specimen embedded. The tube containing beads, resin, and sample was stored in a dark place for 2–3 d till it was solidified for LSFM imaging. For the cell body‐based registration of brain block, the sample was directly embedded in the resin without the procedure of mixing bead. For whole brain imaging, the brain was dissected with keeping an ≈5 mm long spinal cord (Figure 3a). After optical clearing, the sample could be mounted to the stage via connecting the hardened spinal cord with the beam shaft of the rotating motor (Figure 3a).

*Multiview Imaging Acquisition*: The brain samples were scanned under eight views. Each times of scanning was executed along a nonaxial direction with a step‐size significantly smaller than the thickness of light sheet. Under continuous scanning mode, this value was determined by the scanning velocity and camera frame rate, varying from 0.3 µm (for brain block) to 1 µm (for whole brain), depending on the optical configuration. The corresponding acquisition time for total eight views was around 20 and 30 min, respectively. The high‐magnification SPIM images for comparison were obtained using 20× objective plus a thinner light sheet of ≈6.5 µm. Finally, hundreds of gigabyte raw images were transferred to a high‐capacity solid‐state drive redundant array of independent disks of the workstation via the camera link cable.

*SVR‐MVD Reconstruction*: SVR computation combined multiview Bayesian deconvolution^18^ was implemented to achieve isotropic high‐resolution reconstruction of whole mouse brain. Under each view, a series of low‐resolution image stacks were extracted from the oversampled raw data.^17^ All low‐resolution stacks were correlated with each other in term of subvoxel‐resolution displacements and spatially registered to a high‐resolution image stack with an oblique, subvoxel shift. The multiple low‐resolution images and an initial guess of high‐resolution image were input into a maximum‐likelihood‐estimation based computation model to iteratively obtain a converged high‐resolution image. This high‐resolution estimate was corrected into the final reconstruction by a voxel realignment, which recovers the accurate shape of the sample. In practice, the SVR algorithm was applied in parallel to quickly obtain resolution‐enhanced results for all the views.

After SVR processing for each view, the resolution‐enhanced results were regarded as input for multiview reconstruction in Fiji program. Similar with the bead‐based registration method,^14^ here the neuron cell bodies were recognized as fiducial makers to establish the correspondences between each two views. Then all the SVR views could be precisely registered using these correspondences. A multiview Bayesian deconvolution^14^ was applied at the final step to rationally gather the information from all the registered SVR views and generate an output image with containing complete sample information as well as enhanced isotropic resolution. An improved Richardson–Lucy deconvolution was used to obtain the final deconvolved image with faster convergence. Furthermore, this SVR‐MVD computation procedure could be highly parallelized with GPU‐based acceleration. The whole processing time for an entire brain atlas (400 gigavoxels) was ≈12 h on a workstation equipped with dual E5‐2630 CPU, quad Geforce 1080Ti GPU and 1 TB memory. This time consumption could be further reduced with employing more powerful computation units.

*Confocal Microscope and Ultramicroscope Imaging*: The confocal images are taken by Olympus FV3000 under 10× objective (UPLSAPO10X/0.4 NA), with a voxel size of 0.8 µm × 0.8 µm × 2 µm at 0.5 Hz (Figures S9–S11, Supporting Information). The whole brain images taken by commercial light‐sheet microscope (UltraMicroscope, LaVision BioTec) are acquired at 1.6× and 8× magnification (MVPLAPO 2 XC/0.5 NA with 0.8 and 4 zoom ratio, respectively), which take about 20 and 450 min, respectively (Figure S14, Supporting Information).

*Software*: The synchronization of scanning and acquisition was accomplished by LabVIEW (National Instruments). The SVR processing was implemented by customized code and computed with compute unified device architecture acceleration (https://github.com/Fei-Lab/subvoxel-resolving-algorithm). The multiview registration was processed in Fiji. The planar *x*–*z* planes of PSFs were performed using ImageJ. The 3D rendering of PSFs was visualized by Amira (Visage Imaging). The visualizations of neuron imaging, including planar projections, 3D renderings, neuron tracing, were performed by Imaris (Bitplane).

## Conflict of Interest

The authors declare no conflict of interest.

## Author Contributions

J.N., S.L., and T.Y. contributed equally to this work. P.F. and D.Z. conceived the idea and oversaw this study. J.N., S.L., and T.Y. performed the experiment. Y.L., P.W., and Y.H. prepared the sample. J.N., S.L., J.P., and F.Z. processed the data. J.N., S.L., D.Z., S.Z., W.M., and P.F. analyzed the data and wrote the manuscript.

## Supporting information

Supporting Information

Supplemental Video 1

Supplemental Video 2

Supplemental Video 3

Supplemental Video 4

Supplemental Video 5
