# Supplementary material for: Fast, 3D Isotropic Imaging of Whole Mouse Brain Using Multiangle‐Resolved Subvoxel SPIM
Source: Adv Sci (Weinh). 2019 Dec 3;7(3):1901891. doi: 10.1002/advs.201901891 (PMC7001627; doi:10.1002/advs.201901891)
Supplement: Supplementary file 1 — Supporting Information [file ADVS-7-1901891-s001.pdf]

Copyright WILEY-VCH Verlag GmbH & Co. KGaA, 69469 Weinheim, Germany, 2019.

ADVANCED SCIENCE
Open Access

Supporting Information

for *Adv. Sci.,* DOI: 10.1002/advs.201901891

Fast, 3D Isotropic Imaging of Whole Mouse Brain Using Multiangle-Resolved Subvoxel SPIM

Jun Nie, Sa Liu, Tingting Yu, Yusha Li, Junyu Ping, Peng Wan, Fang Zhao, Yujie Huang, Wei Mei, Shaoqun Zeng, Dan Zhu, and Peng Fei**

Supporting Information

Fast, 3D isotropic imaging of whole mouse brain using multi-angle-resolved subvoxel SPIM

Jun Nie[+], Sa Liu[+], Tingting Yu[+], Yusha Li, Junyu Ping, Peng Wan, Fang Zhao, Yujie Huang, Wei Mei, Shaoqun Zeng, Dan Zhu[*] and Peng Fei[*]

J. Nie, S. Liu, J. Ping, F. Zhao, Prof. P. Fei
School of Optical and Electronic Information, Huazhong University of Science and Technology, Wuhan, 430074, China
E-mail: feipeng@hust.edu.cn
Dr. T. Yu, Y. Li, P. Wan, Prof. S. Zeng, Prof. D. Zhu
Britton Chance Center for Biomedical Photonics, Wuhan National Laboratory for Optoelectronics, Huazhong University of Science and Technology, Wuhan, 430074, China
E-mail: dawnzh@mail.hust.edu.cn
Dr. T. Yu, Prof. D. Zhu, Prof. S. Zeng
MoE Key Laboratory for Biomedical Photonics, Huazhong University of Science and Technology, Wuhan, 430074, China
Y. Huang, Prof W. Mei
Department of Anesthesiology, Tongji Hospital, Tongji Medical College, Huazhong University of Science and Technology, Wuhan, 430030, China

Supplementary Note 1: The Mars-SPIM imaging

The Mars-SPIM is derived from standard SPIM[1], which illuminates the sample with a planar laser-sheet and collects the fluorescence with a wide-field detection. The differences are Mars-SPIM uses a non-axial scan instead of axial z scan[2], and at the same time applies a continuous oversampling instead of stepwise Nyquist sampling. Under each angle of view, the small nonaxial step generates sub-pixel shifts s_x, s_y, s_z in x, y, z axes simultaneously among the adjacent frames. These sub-voxel-size shifts can be calculated by the non-axial scanning step s and the oblique angle θ as following:

$$s_x = s_y = s * \sin\theta \tag{1-1}$$

$$s_z = s * \sqrt{1 - 2\sin^2\theta} = s * \sqrt{s * \cos 2\theta} \tag{1-2}$$

The group number n of LR can be calculated by

$$n = pixel/s_x \tag{1-3}$$

Where *pixel* means the lateral pixel size of each slice, which is given by the camera pixel size divided by the magnification. The value of n should be set close to the total enhancement factor (E), to avoid the under- or over-determined computation. Finally, as we finish the entire scanning distance d, we obtain $N = d/s$ frames of raw images, which are encoded with quantified high-frequency spatial shifts for the following SVR-MVD computation.

The native lateral and axial resolutions of the system are determined by the detection and illumination, separately. It is noted that, in Gaussian type light-sheet microscopy, there is a trade-off between high axial resolution (small value) and large confocal range (large value), described as

$$z_{confocal} = \frac{2*(0.85)^2\pi(z_{FWHM})^2}{\lambda} \quad (1\text{-}4)$$

It means when using light-sheet to illuminate large samples, e.g. whole organs, the axial resolution has to be compromised. However, in Mars-SPIM, this issue is circumvented by the SVR-MVD procedure, which computationally improves the axial resolution over ten folds, equivalent to 100 times extension of confocal range. Therefore, it is capable of reconstructing large samples at high spatial resolution.

Supplementary Note 2: SVR

LR image extraction

The original oversampled images Y are then divided into multiple sets of LR image stacks. We extract by one-third of the light sheet thickness ***l***. The LR stacks can be described as:

$$L_k = Y\left(\frac{(k-1)*l}{3*s_z} + n\right) \quad (2\text{-}1)$$

$$k = 1,2,3 \ldots, D_z$$

Where D_z is z dimension of LR images (frame number)

$$D_z = [\frac{N*s_z*3}{l}] \quad (2\text{-}2)$$

The generated LR stacks, L_k, are similar to each other with a sub-voxel shift.

Image degradation model

The core idea of the super-resolution algorithm is to obtain the final enhanced high resolution (HR) image from a series of spatially-correlated, low-resolution and large-FOV images by the sub-voxel-resolving algorithms, to super-resolve high-resolution details across the entire large samples.

We perform the sub-voxel-resolving process using the following model[3]:

$$Y_k = D_k H_k F_k X + V_k \qquad \mathrm{k} = 1, \ldots, \mathrm{N} \tag{2-3}$$

Where F_k is the warping operator, H_k is blurred operator by continuous point spread function (PSF), D_k is downsampling operator by CCD and V_k is the added system noise. Y_k is the acquired image.

Initial guess

The SVR procedure starts from the initial guess X_1 , which is simply the interpolation of one set of LR images

$$X_1 = Interpolate(L_1, [D_{hr\_x}, D_{hr\_y}, D_{hr\_z}]) \tag{2-4}$$

$$D_{hr\_x} = D_x * e_x \tag{2-5}$$

$$D_{hr\_y} = D_y * e_y \tag{2-6}$$

$$D_{hr\_z} = D_z * e_z \tag{2-7}$$

Where $e_x\, e_y, e_z$ are the enhancement factors in three directions of x, y, z, respectively. $D_{hr\_x}, D_{hr\_y}, D_{hr\_z}$ and $D_x, D_y,\ D_z$ represent the dimensions of each HR and LR stack. The total enhancement factor can be described as $E = e_x * e_y * e_z$.

Iterative optimization

The multiple low-resolution sequences $L_k(z)$ and the initial guess of high-resolution images $G_0(z)$ are then input into a maximum-likelihood-estimator to iteratively get the converged solution of X, which is the final high-resolution image stack.

$$X = ArgMin(\sum_{k=1}^{N} \rho(L_k, D_k H_k F_k \bar{L}) + \lambda\gamma(\mathrm{X})) \tag{2-8}$$

The regularization part $\lambda\gamma(\mathrm{X})$ is added to this ill-posed problem, as a constraint of the possible solution to effectively accelerate the convergence. Then by a steepest descent method, the estimate from the $(i+1)_{th}$ iteration can be inferred as

$$X_{i+1} = X_i - \beta \sum_{k=1}^{N} L_k \qquad (2\text{-}9)$$

Realignment

Due to the non-axial scanning strategy, the raw HR estimate X_{i+1} exists a distortion compared to the ground truth. According to the oblique scanning angle , the program recovers the HR X_{i+1} into the accurate shape of the sample via a voxel re-alignment, generating the high-resolution, accurate, sub-voxel-resolved image X as final output.

$$X = reshape(X_{i+1}) \qquad (2\text{-}10)$$

Supplementary Note 3: Multi-view deconvolution

With SVR applied to the LSFM images of all the views, we first obtain the resolution-enhanced images under all the views. For each view, the image resolution has been better balanced with the large FOV. However, these single-view volumetric images remain anisotropic and degraded by deep tissue scattering. We then perform multi-view deconvolution (MVD), a technique well-known with isotropic, complete visualization of thick samples, on the intermediate SVR images, to furthermore obtain an isotropic super-resolution output with fusing all the scattering-free information from multiple views.

Segmentation of image feature points

We select incorporate fiduciary markers of image feature for sample independent registration. For brain block, we uniformly mix the sample with fluorescent microspheres and dope them in rigid transparent resin which is a packaging material for fixing the clarified brain sample. For whole brain sample, we successfully use the neuron cell bodies as marker for precise registration. All the beads are detected with high accuracy using 3D Laplace filter ∇^2.

Establishing correspondences

After identifying the interest points, the MVD program creates translation and rotation invariant geometric descriptor, which contains one bead and its three-nearest neighbors in 3-D space, to define the correspondence among different views. An affine transformation mode is then applied to substantially create the correspondences with optimal registration.

Globally minimized the displacement

MVD procedure defines an affine transformation T_{AB}, which maps view A and B using least squares method, to define the error between two views.

$$argmin_{T_{AB}} \sum_{(\vec{a},\vec{b})\in C_{ab}} \left\| T_{AB}\vec{a} - \vec{b} \right\|^2 \quad (3\text{-}1)$$

Bayesian-based deconvolution

After multiple views are registered, the following Bayesian-based deconvolution[4] is applied at the final step to rationally gather information from all the views and compute an estimate with isotropic axial resolution and improved contrast. Bayesian-based deconvolution algorithm first models all views of image and PSF as a Bayesian probability distribution, iteratively seeking a maximized probability result based on the multi-view images. The solving process can be described as following equation:

$$\psi^{r+1}(\xi) = \psi^{r}(\xi) \int_{x} \frac{\Phi_{v}(x_v)}{\int_{\xi} \psi^{r}(\xi) P(x_v|\xi) d\xi} P(x_v|\xi) dx_v \quad (3\text{-}2)$$

Where $\psi^{r}(\xi)$ is the deconvolved image at iteration r, $\Phi_v(x_v)$ is the input views, $P(x_v|\xi)$is the probability of a measurement (PSF) of each view.

The final derivation and optimization under multi-view image can be expressed as:

$$\psi^{r+1} = \psi^{r} \prod_{v\in V} \frac{\Phi_v}{\psi^{r} * P_v} * \prod_{v,\ w\in W_v} P_v^{*} \quad (3\text{-}3)$$

The detailed implementation of bead-based registration and MVD methods can be furthermore found in Nature Method paper by Stephan Preibisch etal[5].

Supplementary Note 4: Determining the number of views

In order to counterbalance the acquisition time and the effect of SVR-MVD, we carefully seek the optimal number of views for whole brain imaging. The SVR-MVD results processed by one, two, four, and eight views are compared in Figure S7. Through comparison, it is quite convincing that four views are the minimal requirement to obtain a basically isotropic resolution enhancement. However, eight views result still shows higher contrast and richer details in three dimensions. Given the fact that imaging and post-computation time both increase approximately linear with the increase of the number of views, eight-views acquisition most likely fights the best balance between the temporal and spatial resolution for Mars-SPIM.

Supplementary Note 5: Throughput, photobleaching and SNR measurements

5.1 Throughput:

Imaging throughput represents how much optical information a system can extract from the sample over time. Thus, it's an important factor for evaluating an optical system. For a 3D imaging modality, its throughput (*T*) can be calculated by the following equation:

$$T = \frac{image\ volume}{voxel\ size \times image\ time}\ (voxel/second) \qquad (5\text{-}1)$$

Here we use a 3.3 × 3.3 × 2 mm brain tissue sample to compare the throughput of various imaging modes demonstrated in this work. In conventional SPIM (4×), the acquisition time are about 80 seconds under standard stepwise scanning mode. With a 1.625 × 1.625 × 8 μm voxel, the throughput can reach up to 1.2×10^7 voxel/second. As for higher-magnification SPIM at 20×, it takes 3 hours to obtain 36 tiles that cover the entire sample. With a smaller voxel size of 0.325 × 0.325 × 2 μm, the throughput is calculated 9.4×10^6 voxel/second. For confocal experiment (Olympus FV3000, 10× objective), due to the point-scan mode, this value is unsurprisingly down to 5×10^5 voxel/second. In contrast, Mars-SPIM takes 2000 seconds to acquire total eight views of raw data. With a super-resolved isotropic voxel size of

0.4 × 0.4 × 0.4 μm, the equivalent throughput of our method can be as high as 1.7 × 10^8 voxel/second. The image results by these methods and their throughput comparison are shown in Supplementary Figure 9 and Table 1.

5.2 Photobleaching rate and SNR

A selected volume was repetitively imaged 12 times to compare the photobleaching rates of 4× SPIM, 20× SPIM (stitching), 10× confocal microscope and Mars-SPIM. The fluorescence variation in a 100 μm × 100 μm × 100 μm sub-volume was then calculated for each method by: (ⅰ) applying a mask to extract only the top 1% brightest voxels; (ⅱ) calculating the average intensity of these brightest voxels; (ⅲ) Normalizing the signals according to the initial stack for each method. The bleaching rate was finally plotted over the time for each method in Supplementary Figure 9.

With knowing the signal intensity, the SNR (Supplementary Figure 7 and 10) can be obtained as well simply by: (ⅰ) calculating the average values of 90% darkest voxels to obtain the noise; (ⅱ) dividing the signal value by noise value to obtain the SNR.

Supplementary Note 6: Radar map analysis

In Figure 2h, we draw a radar map to visualize the lateral-, axial resolutions, imaging speed, system throughput and signal preservation for different methods. We first renormalize these different parameters so that they can be compared together. For signal preservation rate (percentage) and imaging speed (volume/time), we just normalize the values linearly. For the lateral and axial resolutions, we normalize their reciprocal - it hence means a larger value shows a better spatial resolution. For the throughput, the huge variation range is narrowed down by normalizing the log of the original values. Finally, along each dimension, the lager value (close to 1) indicates better performance. The detailed values of the radar map are further shown in Table S1.

References

[1] a) J. Huisken, J. Swoger, F. Del Bene, J. Wittbrodt, E. H. Stelzer, Science 2004, 305, 1007; b) J. Huisken, D. Y. Stainier, Development 2009, 136, 1963; c) P. G. Pitrone, J. Schindelin, L. Stuyvenberg, S. Preibisch, M. Weber, K. W. Eliceiri, J. Huisken, P. Tomancak, Nature methods 2013, 10, 598; d) H. U. Dodt, U. Leischner, A. Schierloh, N. Jahrling, C. P. Mauch, K. Deininger, J. M. Deussing, M. Eder, W. Zieglgansberger, K. Becker, Nature methods 2007, 4, 331.

[2] P. Fei, J. Nie, J. Lee, Y. Ding, S. Li, H. Zhang, M. Hagiwara, T. Yu, T. Segura, C.-M. Ho, Advanced Photonics 2019, 1, 016002.

[3] a) M. Elad, Y. Hel-Or, IEEE Transactions on Image Processing 2001, 10, 1187; b) S. Farsiu, M. D. Robinson, M. Elad, P. Milanfar, IEEE transactions on image processing 2004, 13, 1327.

[4] S. Preibisch, F. Amat, E. Stamataki, M. Sarov, R. H. Singer, E. Myers, P. Tomancak, nature methods 2014, 11, 645.

[5] S. Preibisch, S. Saalfeld, J. Schindelin, P. Tomancak, Nature methods 2010, 7, 418.

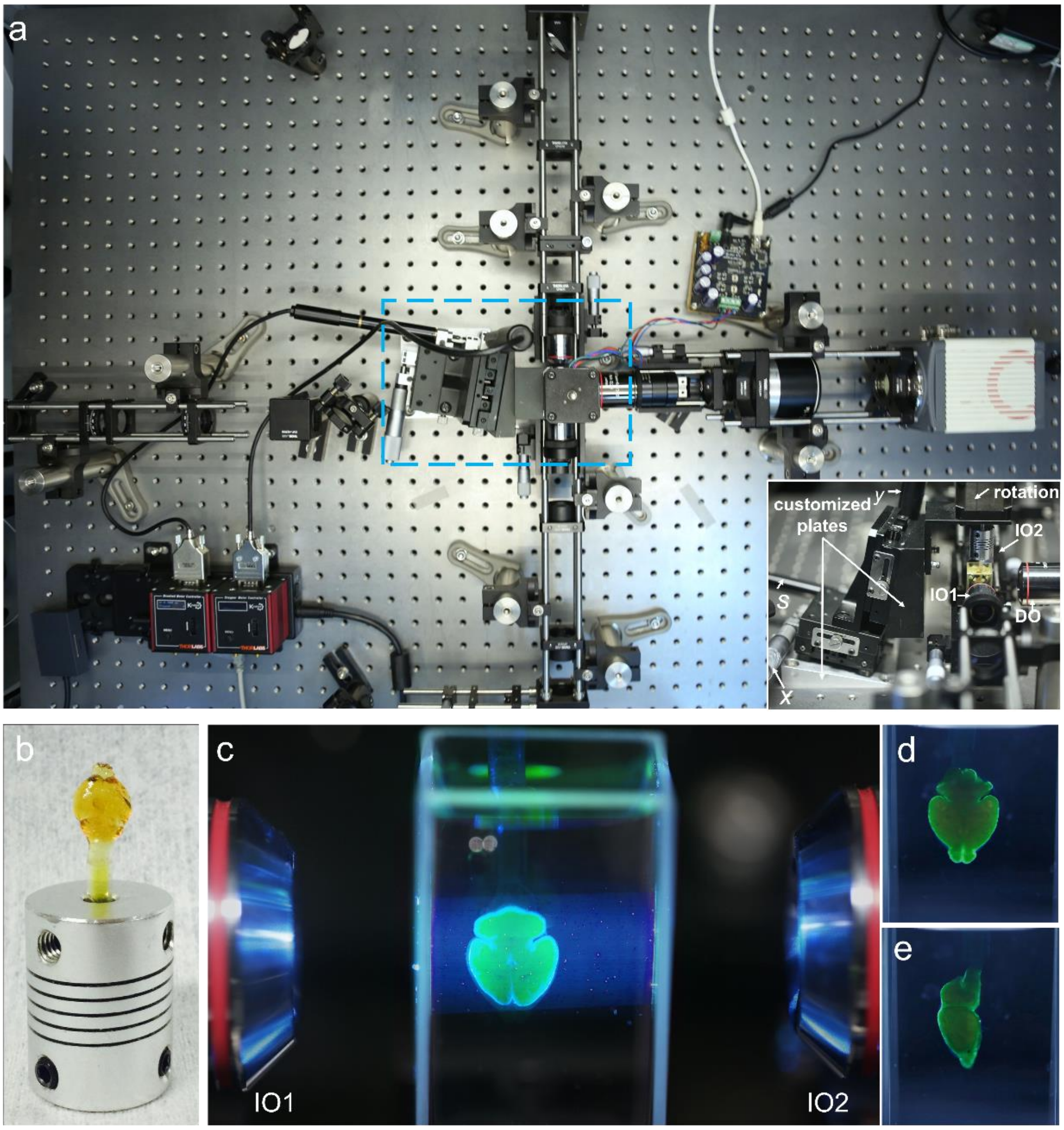

Supplementary Figure 1. Mars-SPIM system. (a), the photograph of our home-built Mars-SPIM system. It is a SPIM system with simple retrofit added. The system contains a specialized 4-degrees-of-freedom stage, which is comprised of three-axis sample translation along *x*, *y* and oblique scanning *s* directions, and sample rotation around y axis, as shown in the insert. **(b)**, the photograph of the cleared whole brain sample, which is mounted on a rotation shaft coupler via clamping the harden spine cord. **(c)-(e)**, The close-up views of the whole-brain Mars-SPIM imaging. The cleared whole brain is illuminated plane by plane using a 488 nm laser-sheet. The excited green fluorescence signals of the planes are sequentially

collected by the orthogonal wide-field detection. The sample is rotated by step motor and imaged under multiple views.

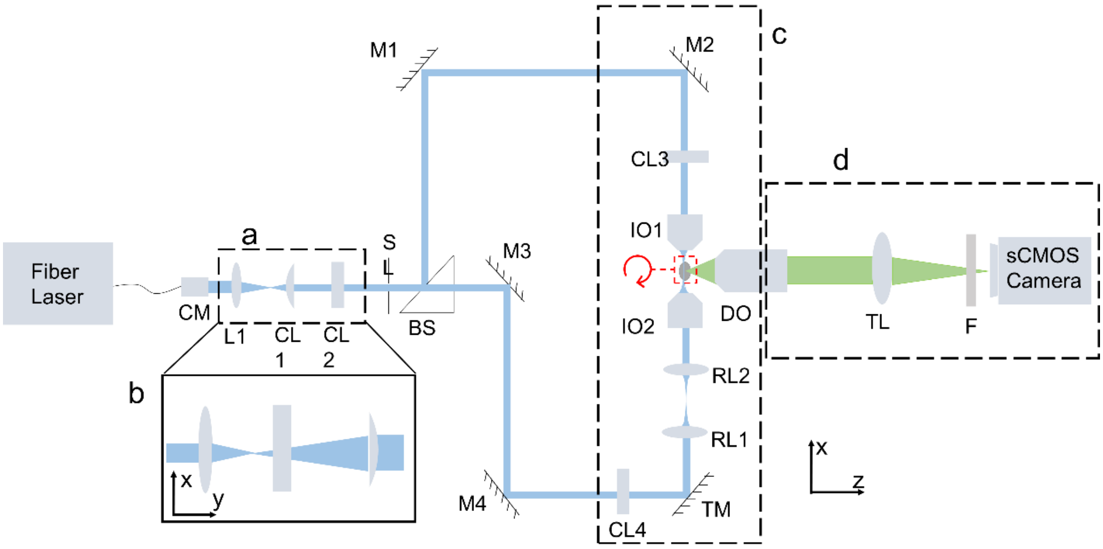

Supplementary Figure 2. Optical layout of Mars-SPIM. First, the light from fiber laser is collimated and expanded. Box **(a)** and **(b)** shows the expansion of the light along y axis, modulating the round-shape beam into an elliptic-shape one by using the sandwich combination of one doublet lens (f_0 = 50 mm) and two cylindrical lenses (f_1 = 30 mm, f_2 = 150 mm). This elliptic-shape beam is well suited for the generation of large scale laser-sheet, which can illuminate the entire brain at one time. Then the beam is split into two opposite parts and reflected by mirrors to excite the sample from dual sides. Box **(a)** and **(b)** shows the tunable dual-side illumination path and the wide-field detection, respectively. Abbreviations: CM, collimator; L, doublet lens; CL, cylindrical lens; SL, adjustable slit; M, mirror; RL, relay lens; IO, illumination objective; DO, detection objective; TL, tube lens; F, filter.

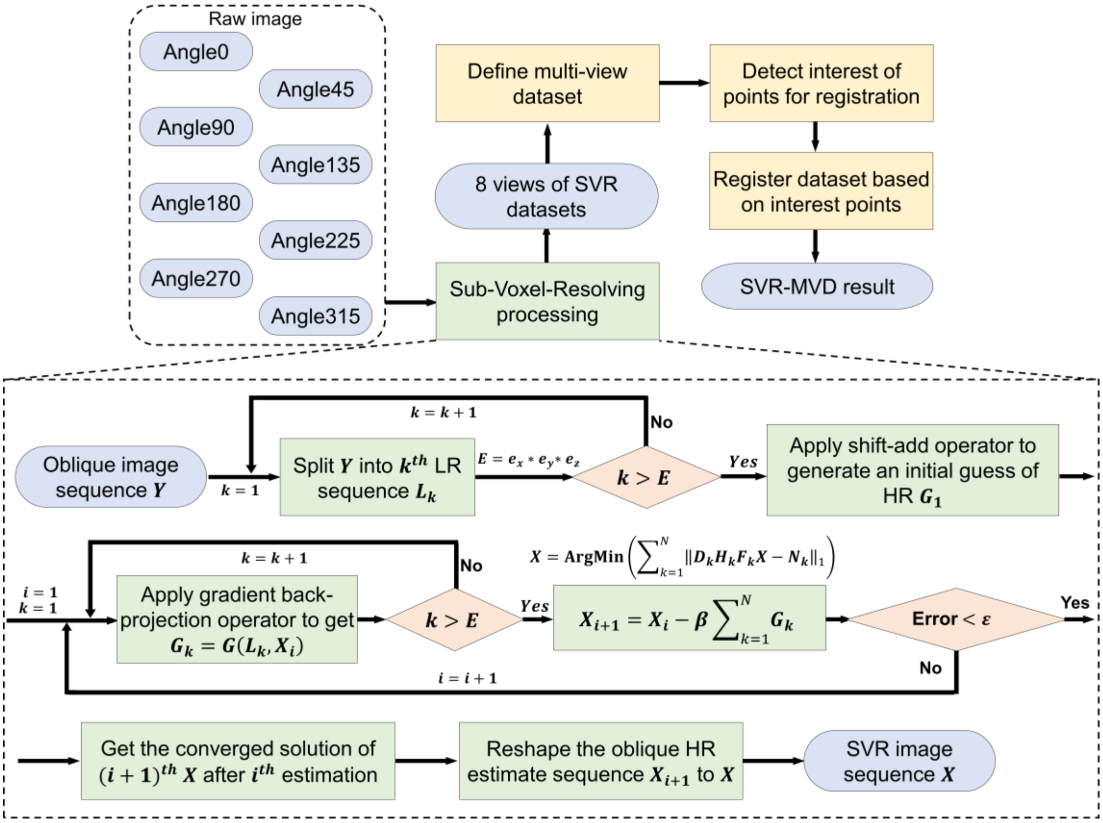

Supplementary Figure 3. Flow chart of SVR-MVD procedure. Eight views of raw images are first processed by sub-voxel-resolving (SVR) algorithm. At SVR step, the original oblique image sequence Y encompassing an ultrafine step-size, is split into k groups of low resolution stacks L_k with voxel depth being a half of light-sheet thickness (according to Nyquist sampling principle). Then the multiple low-resolution stacks L_k and the initial guess of high-resolution image G_1, are inputted into a shift-based maximum-likelihood-estimation to iteratively obtain the converged solution of X_{i+1} after i iterations computation, at which the X_{i+1} shows very little difference with X_i via a steepest decent comparison. Finally, the high-resolution estimate X_{i+1} is recovered into the accurate shape of the sample by a voxel re-alignment, generating X as final output. With having the sub-voxel-resolved X for all the eight angles of views, they are furthermore used as input for multi-view deconvolution in Fiji[5].

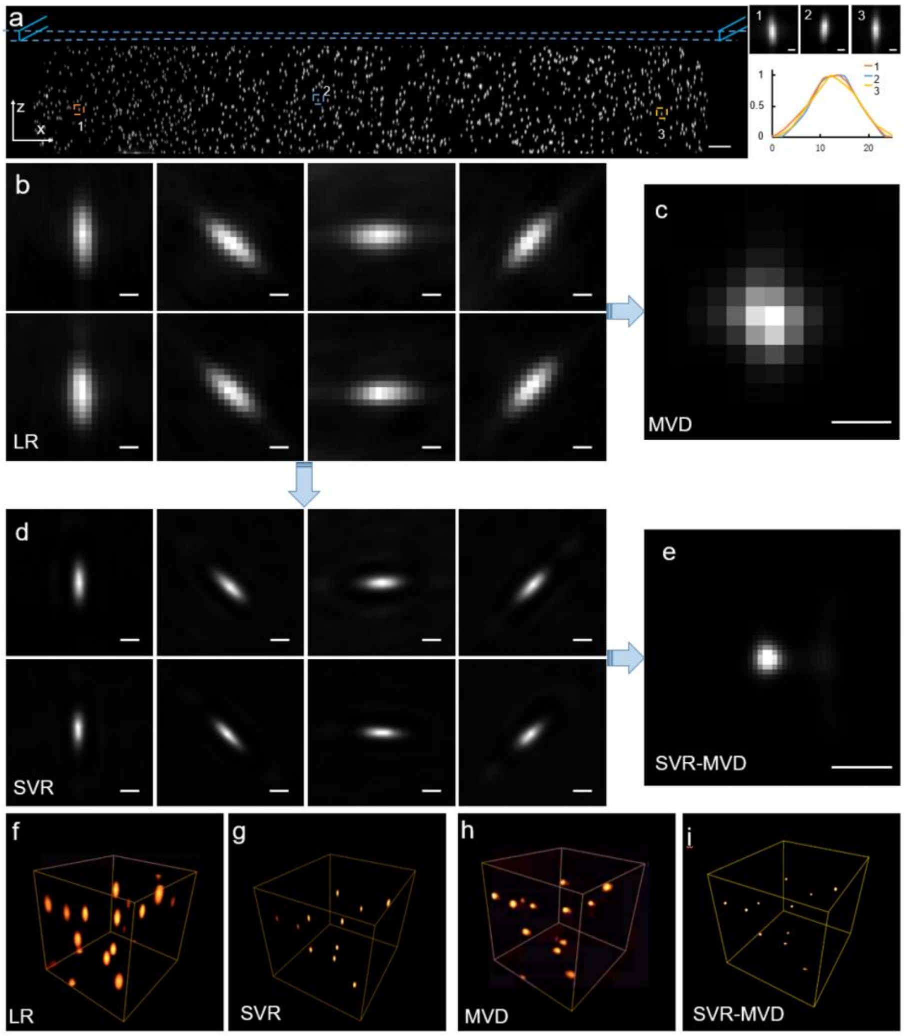

Supplementary Figure 4. point-spread functions (PSFs) of SPIM, mvd-SPIM and Mars-SPIM. (a), The x-z point spread function (PSF) images of fluorescent beads (~500 nm) obtained by 4×/0.16 detection objective plus 12 μm thick laser-sheet illumination. Three resolved beads across the FOV (x direction) are slected to show their uniform axial extents (1,2,3). Their line profiles are further ploted to quatitatively charecterize the uniform light-sheet illumination of ~12 μm thickness (FWHM). **(b)**, PSFs of registered LR views at angle 0°-315° (45°interval for each view). **(c)**, PSFs of the registered SVR views. **(d)**, PSF of conventional MVD based on eight-LR views. **(e)**, PSF of SVR-MVD based on eight-SVR views. **(f)**, 3-D rendering of the PSFs, by LR, SVR, conventional MVD and SVR-MVD. The PSFs in **(a)**-**(e)** are all shown in x-z planes. Scale bars: 100 μm in **(a)**, 5 μm in insets of **(a)** and **(b)-(e)**.

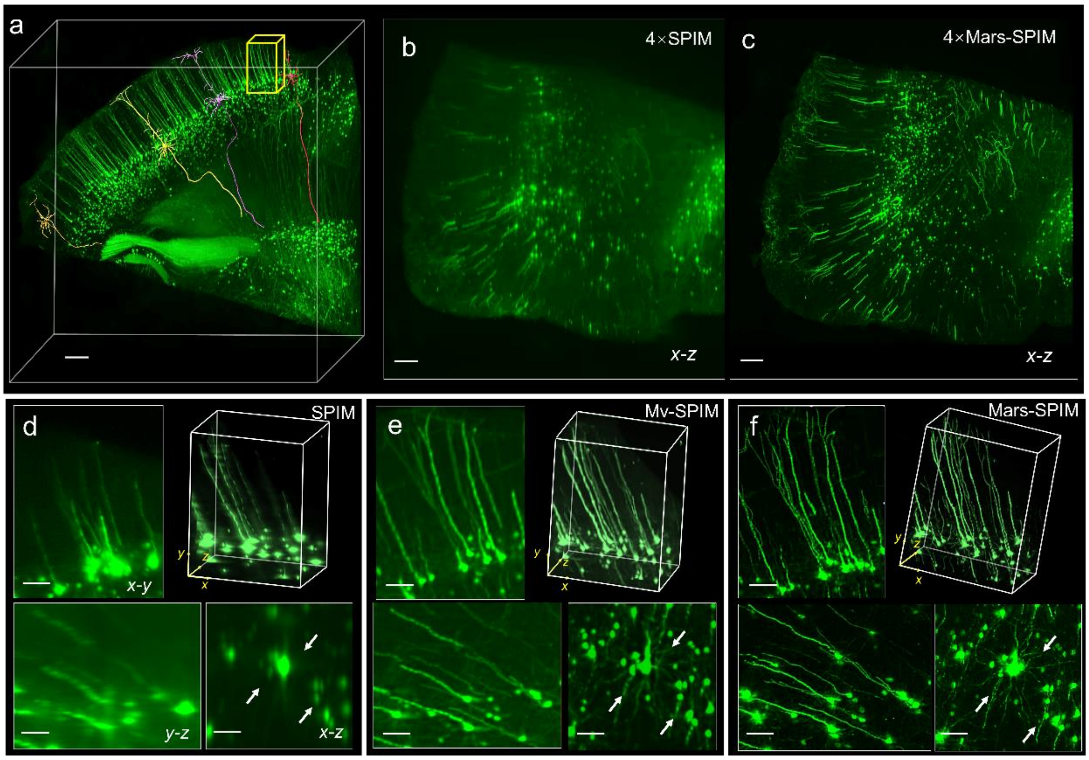

Supplementary Figure 5. Mars-SPIM imaging of brain blocks using bead based registration. The optical resin (D.E.R. 332: D.E.R. 736: IPDA=11.5:3.5:3, by volume) homogeneously mixed with fluorescence beads (~500 nm) is formulated to package the brain block. Then the embed brain sample together with the surrounding beads are imaged under 8 views with 4×/0.28 objective. **(a)**, the volume rendering of Mars-SPIM result. **(b)** and **(c)** compare the reconstructed x-z planes of the sample by conventional SPIM (4×) and Mars-SPIM (4×). **(d)**-**(f)**, vignette high-resolution views of a small cortex area (yellow box in **a**) by SPIM, conventional multiview SPIM (Mv-SPIM), and Mars-SPIM, respectively. The arrows in x-z planes show the neuron fibers which can’t be identified or finely resolved by SPIM or Mv-SPIM, indicating the significant information enrichment and resolution enhancement by Mars-SIMP. Scale bars: 200 μm in **(a)**-**(c)** and 50 μm in **(d)**-**(f)**.

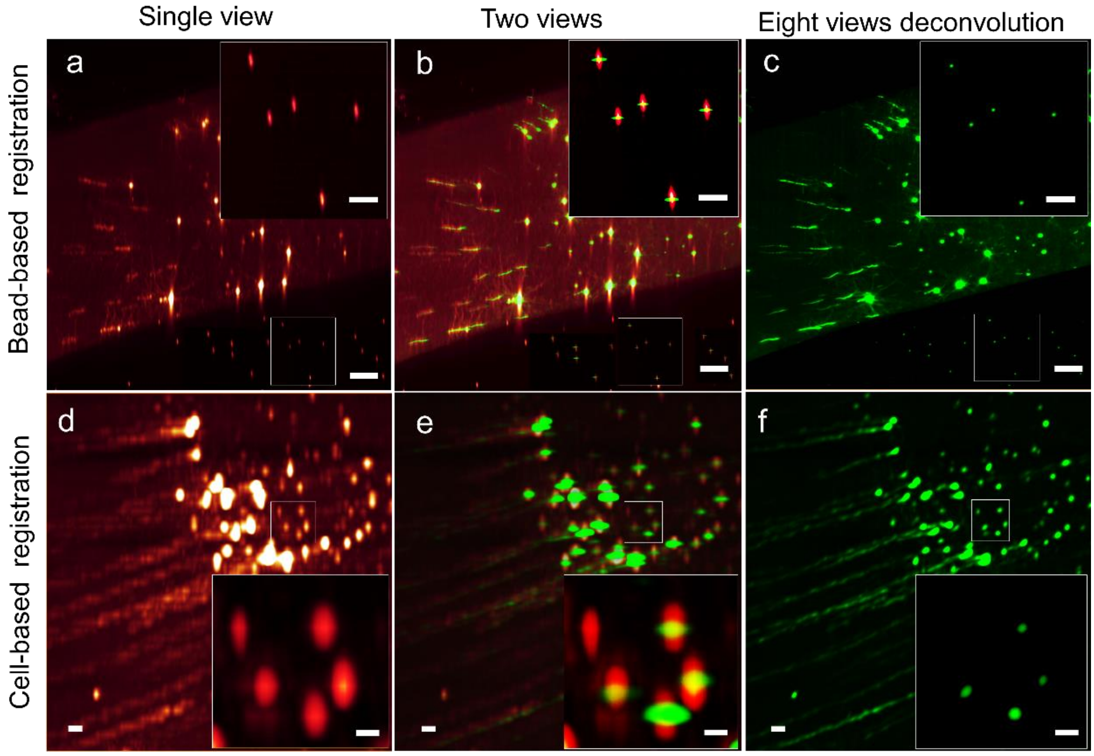

Supplementary Figure 6. Comparison of accuracy by bead-based and neuron cell body-based registration. (a)-(**c**), the results of 0-degree single view, fusion of 0 (red) and 90 degree (green) views using bead registration, and eight-view deconvolution using bead registration, respectively. As compared to **(a)-(c)**, **(d)**-**(f)** correspondingly show the results from cell body based registration, indicating sufficient accuracy achieved for high-resolution reconstruction. Scale bars: 50 μm in **(a)**-**(f)** and 20 μm in the high-resolution inserts.

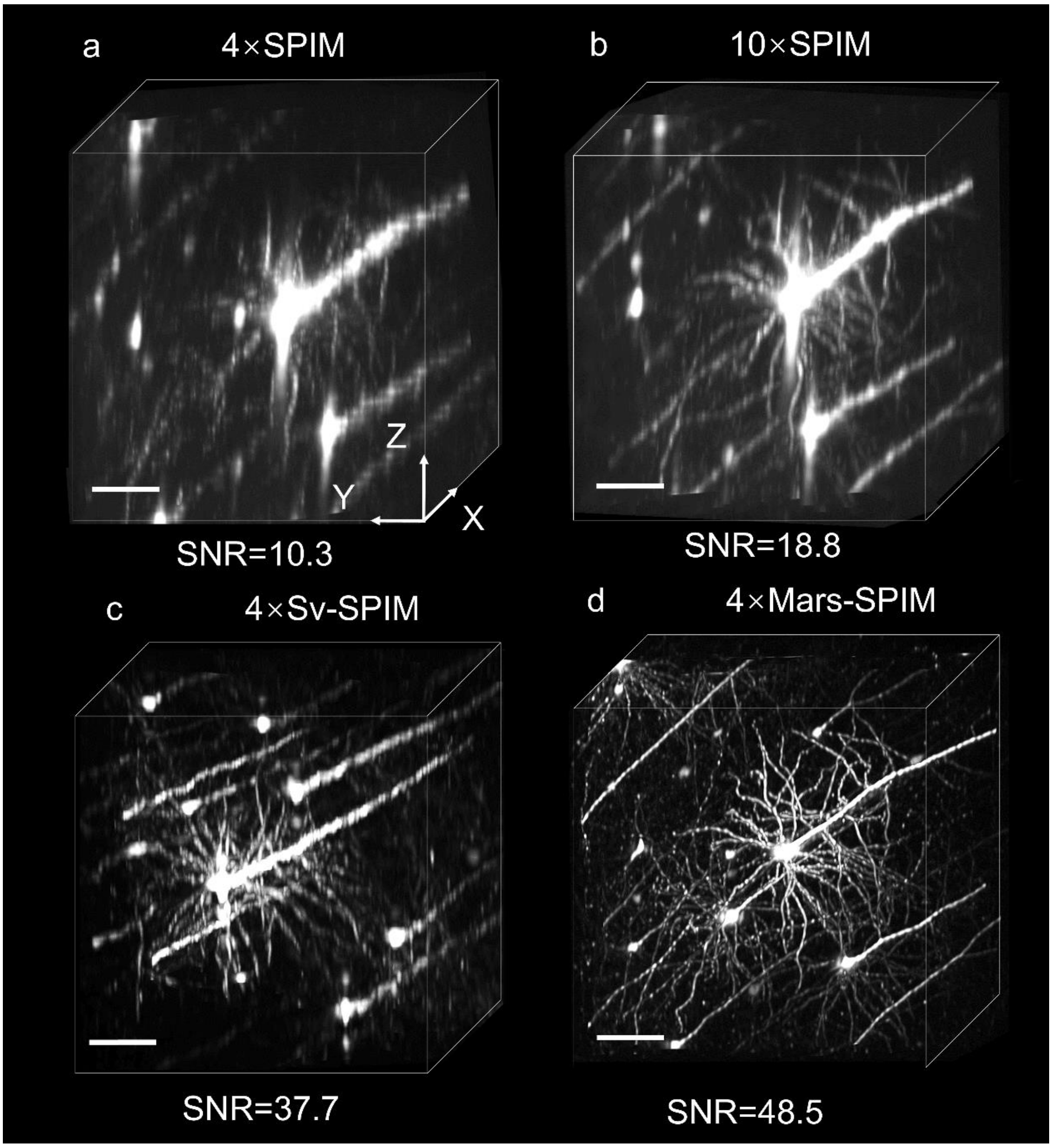

Supplementary Figure 7. Enhancement of signal-to-noise ratio (SNR) by Mars-SPIM. Resolving mouse brain neurons (cortex area) using 4× SPIM (~15 μm laser-sheet, **a**), 10× SPIM (-8 μm laser-sheet, **b**), 4× sub-voxel SPIM (Sv-SPIM) (**c**), and 4× Mars-SPIM (**d**). Besides the significantly improved spatial resolution, Mars-SPIM also shows the highest SNR of 48.5, which is over four times higher than original SPIM image. Sale bars: 50 μm.

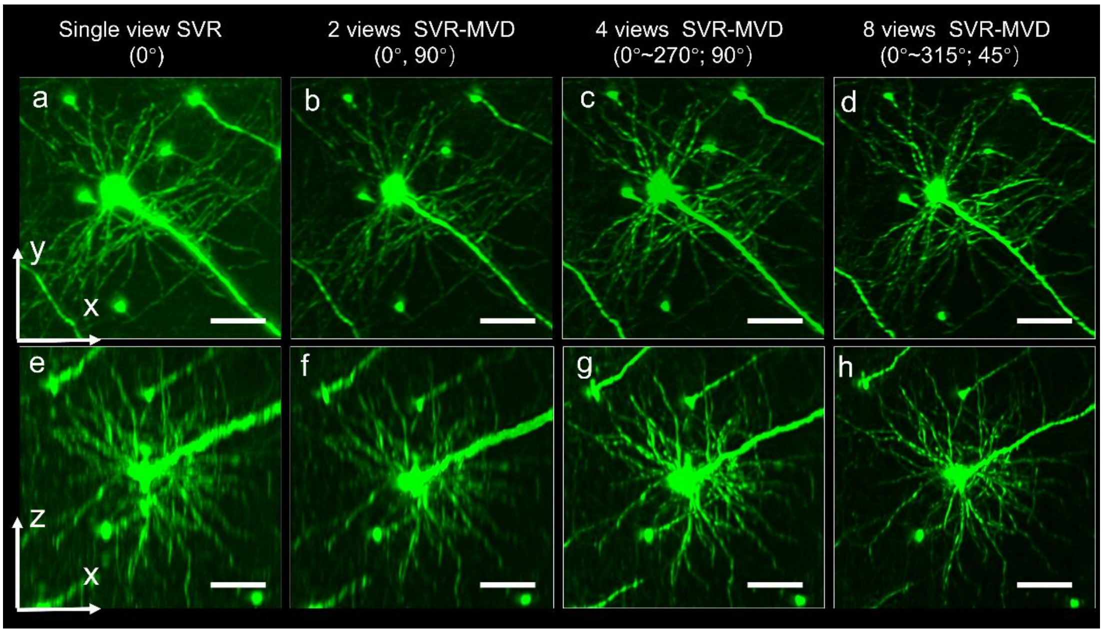

Supplementary Figure 8. SVR-MVD using different numbers of registered views. **(a)-(d)**, the x-y plane results of single view, two views (0˚ and 90˚), four views (every 90˚) and eight views (every 45˚), respectively. **(e)**-**(f)**, the reconstructed x-z plane results. Scale bars: 50 μm.

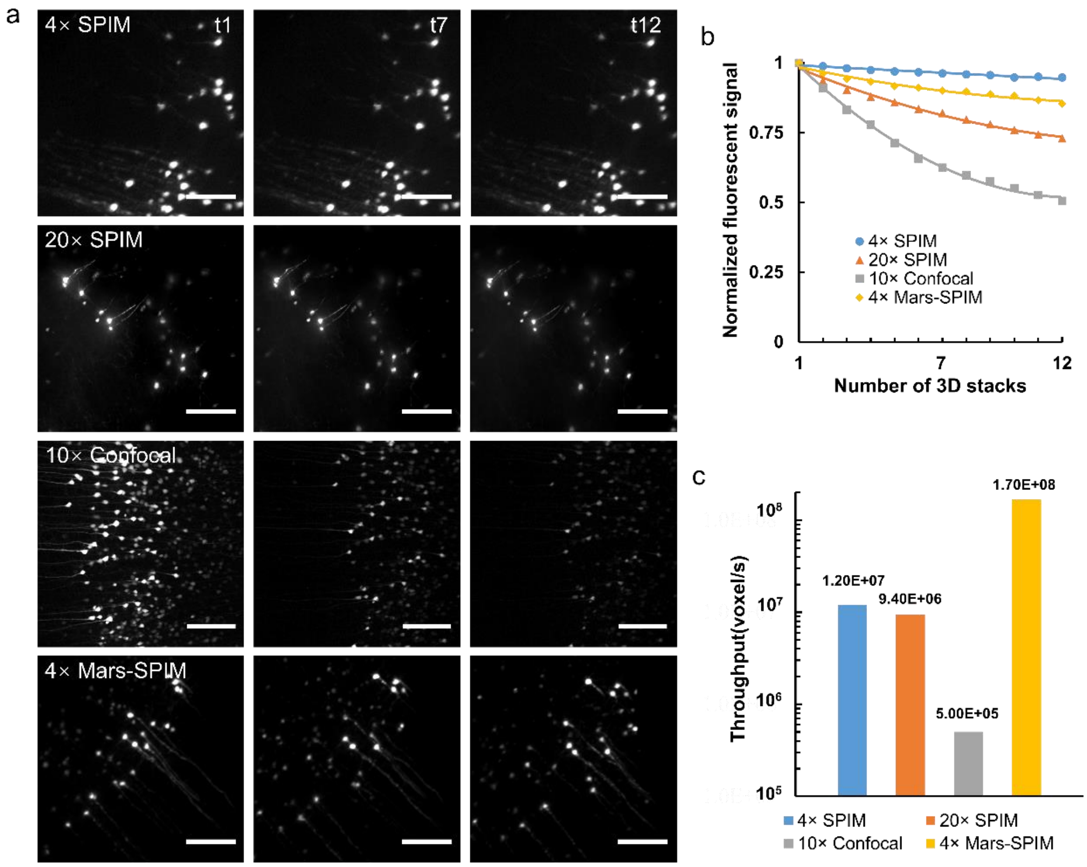

Supplementary Figure 9. Characterization of standard SPIM (4× and 20× stitching), confocal microscope (10×) and Mars-SPIM (4×). For each method, a selected volume was repeatedly imaged for 12 times. **(a)**, signals of the same plane at different time points (t1, t7 and t12). **(b)**, The bleaching rates of fluorescence signals for four methods. **(c)**, the imaging throughputs of four methods. Scale bars: 100 μm in **a**.

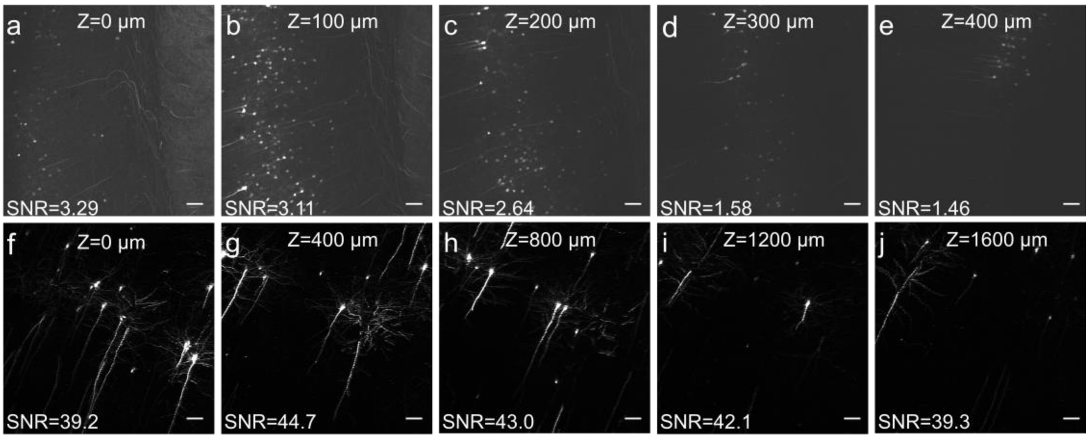

Supplementary Figure 10. Signal-to-noise ratio (SNR) comparison of confocal microscope and Mars-SPIM at different z-depth. **(a)-(e)** show the plane images of mouse cortex from 0 to 400 μm depth acquired by Olympus FV3000 using 10×/0.4 objective. The images are displayed with the same dynamic range. The image SNR drops obviously as the signal goes deeper. As comparison, **(f)-(j)** show the reconstructed images from 0 to 1600 μm depth by Mars-SPIM using 4×/0.28 objective. Scale bar: 50 μm.

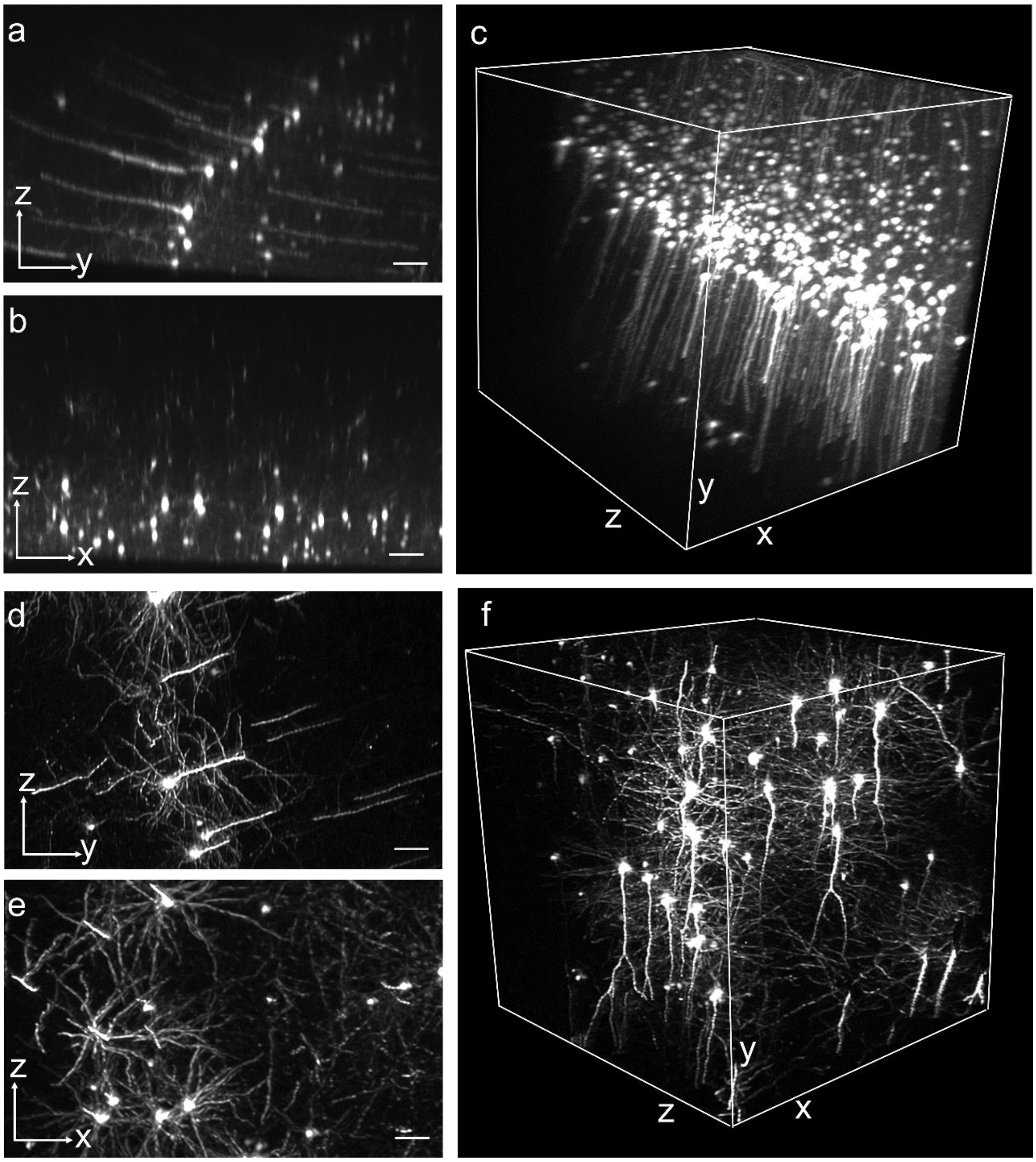

Supplementary Figure 11. Resolution comparison of 10× confocal microscope and 4×Mars-SPIM. (a)-(c) shows the reconstructed xz, yz planes and 3-D reconstruction of mouse cortex acquired by Olympus FV3000 using 10×/0.4 objective. **(d)-(f)** correspondingly shows the results from Mars-SPIM using 4×/0.28 objective. Scale bars: 50 μm.

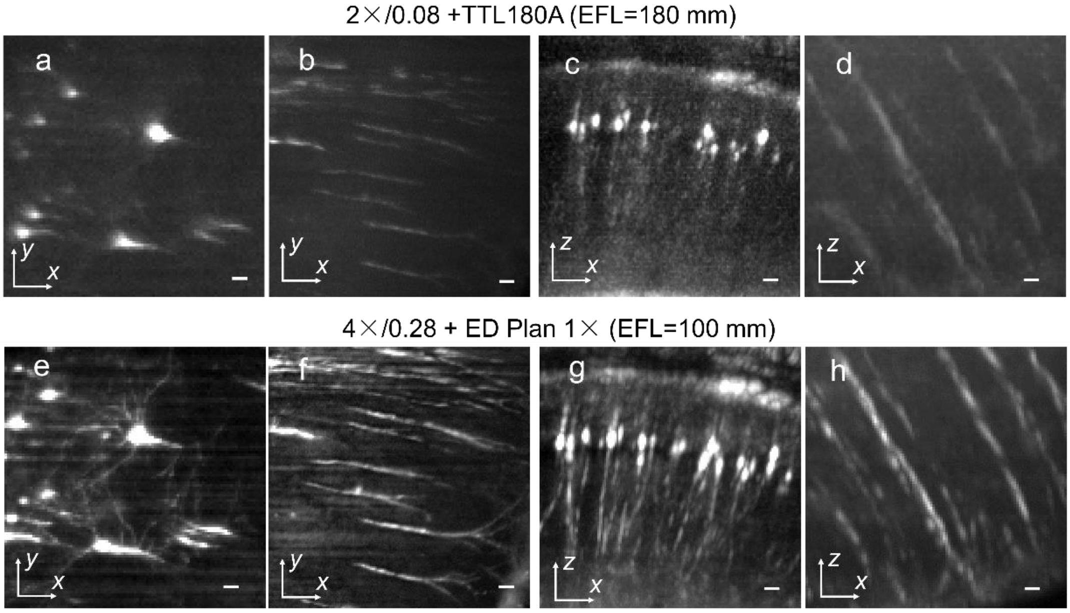

Supplementary Figure 12. Comparing the raw image quality of the conventional and customized detection configurations. Conventional 2× magnification is formed by a 2×/0.08 objective paired with a standard tube lens (TTL-180A, EFL = 180 mm). Due to the small numerical aperture at low magnification, the recorded neuron images show poor SNR (**a-d**), which diminishes the weak signals from the small neuronal fibers. In Mars-SPIM imaging, to preserve the weak signals, we use 4×/0.28 objective (Olympus XLFLUOR4×/340) plus a large-aperture stereo lens (Nikon ED Plan 1×, EFL=100 mm) to form a customized detection path with equivalent magnification of 2.2×. The results from the same brain region are correspondingly shown in **(e)-(h)**. It is quite obvious that by using our customized detection path with larger aperture, Mars-SPIM can detect weak fluorescence from small neuronal fibers. For comparison, the excitation intensity (2.5 w/cm^2) and the exposure time (20 ms) are kept the same for both configurations. Scale bars: 20 μm.

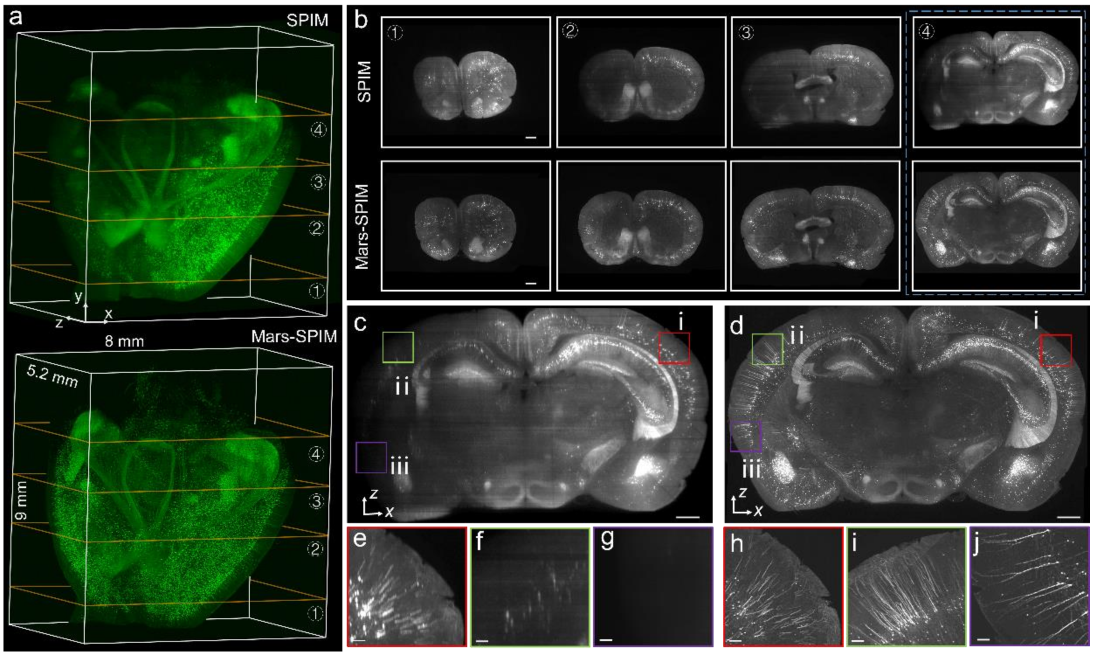

Supplementary Figure 13. The signal recovery and resolution enhancement of whole brain by Mars-SPIM. (a), 3D rendering of reconstructed whole mouse brain, using 2.2×-SPIM and 2.2×- Mars-SPIM, respectively. **(b),** comparison of four coronal planes (x-z) chosen from **(a)**. **(c)-(d),** the magnified views of plane 4. Three regions of interest, which represent near side of laser illumination (right, red box), far side of laser illumination (left, green box), and far-and-deep tissue (bottom, purple box) of the brain, are shown in **(e)-(g)** by SPIM and **(h)-(j)** by Mars-SPIM, respectively. The severely degraded signals at the far side (**f**) and completely lost signals at the far-and-deep tissue **(g)** in SPIM results, are recovered by Mars-SPIM (**i** and **j**). Furthermore, even at the near side where the SPIM can yield its best result **(e)**, Mars-SPIM still shows notable resolution improvement **(h)**. Scale bars: 500 μm in **(b)-(d)** and 100 μm in **(e)-(j)**.

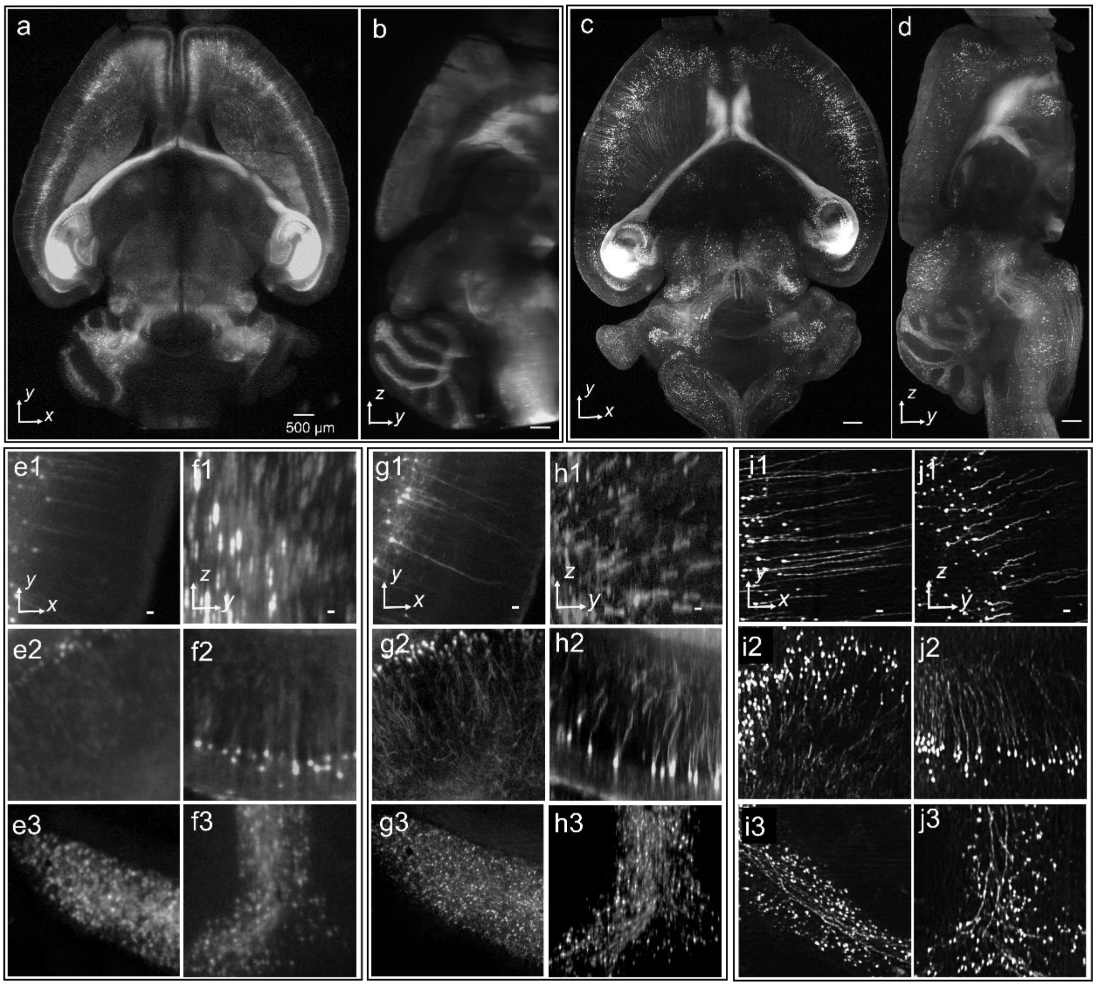

Supplementary Figure 14. Whole mouse brain imaging by commercialized UltraMicroscope (LaVison BioTec) and Mars-SPIM. (a) and **(b)** are the horizontal and coronal planes take by UltraMicroscope under 1.6× (dual-side illumination). Correspondingly, **(c)** and **(d)** are acquired by Mars-SPIM. **e1-f1**, **e2-f2** and **e3-f3** are the xy and xz planes of three small regions selected from **(a)-(b)**. (**g1)-(h1)**, **(g2)-(h2)** and **(g3)-(h3)** shows the results of the same regions acquired by 8× higher magnification of UltraMicroscope. As comparison, **(i1)-(j1)**, **(i2)-(j2)** and **(i3)-(j3)** are the results from the 2.2× Mars-SPIM. The images from UltraMicroscope have been deconvolved to reduce the backgraound blurs. Scale bars: 500 μm in **(a)-(d)** and 20 μm in **(e)-(j)**.

```
Image specific
(FixedImageDimension 3)
(MovingImageDimension 3)
(FixedInternalImagePixelType "short")
(MovingInternalImagePixelType "short")
(Index 0 0 0)
(Spacing 0.125        0.125        0.125)
(Origin 0.0000000000 0.0000000000 0.0000000000)
(Direction 1.0000000000 0.0000000000 0.0000000000 0.0000000000 1.0000000000
0.0000000000 0.0000000000 0.0000000000 1.0000000000)
(UseDirectionCosines "true")
// BSplineTransform specific
(GridSize 22 17 25)
(GridIndex 0 0 0)
(GridSpacing 34.0941794820 35.7527285748 31.1830242843)
(GridOrigin -71.4076668433 -92.1650650143 -34.3310259340)
(GridDirection 1.0000000000 0.0000000000 0.0000000000 0.0000000000 1.0000000000
0.0000000000 0.0000000000 0.0000000000 1.0000000000)
(BSplineTransformSplineOrder 3)
(UseCyclicTransform "false")
// ResampleInterpolator specific
(ResampleInterpolator "FinalBSplineInterpolatorFloat")
(FinalBSplineInterpolationOrder 3)
// Resampler specific
(Resampler "DefaultResampler")
(DefaultPixelValue 0.000000)
(ResultImageFormat "mrc")
(ResultImagePixelType "short")
(CompressResultImage "false")
(OpenCLResamplerUseOpenCL "false")
```

Supplementary Figure 15. Parameter settings in Elastix for registration of Mars-SPIM whole brain to allen brain atlats (ABA).

Supplementary Table 1. The throughput of different methods.

Method	Volume size (mm^3)	Voxel size (μm^3)	Acquisition time (s)	Throughput (voxel/s)
4× SPIM (stepwise)	3.3×3.3×2	1.625×1.625×8	80~	1.2×10^7
4× SPIM (continuous)	3.3×3.3×2	1.625×1.625×8	5~	2×10^8
4× Mars-SPIM	3.3×3.3×2	0.4×0.4×0.4	2000~	1.7×10^8
10× Confocal	3.3×3.3×2	0.82×0.82×2	32000~	5×10^5
20× SPIM	3.3×3.3×2	0.325×0.325×2	11000~	9.4×10^6

Supplementary Table 2. The imaging setups for different samples by different methods

Sample type	Light sheet thickness	Detection objective	Scanning stepsize	Acquisition speed (frames/second)	figure
Fluorescent bead(~500 nm)	12 μm	4×/0.16, Olympus	280 nm	50	Figure 1, Figure S4
Brain block	15 μm	4×/0.28, Olympus	280 nm	50	Figure 2, Figure S3-S11
	6.5 μm	20×/0.45, Olympus	2 μm	3.3	Figure 2, Figure S9
	Confocal, FV3000, Olympus	10×/0.4, Olympus	2 μm	0.5	Figure 2, Figure S9-S11
Whole brain	25 μm	2.2 × (equivalent)	950 nm	50	Figure 3-5, Figure S12-S14
	Ultra-Microscope, LaVison BioTec	1.6×(dual-side illumination)	5 μm	1	Figure S14
		8×	2 μm	3.3	
